# Characterization of dysbiosis patterns in gut microbiota of digestive system cancers: an umbrella review

**DOI:** 10.3389/fmicb.2026.1782471

**Published:** 2026-04-28

**Authors:** Jing Wang, Yingkai Liu, Yan Yu, Xiaoli Chen, Genghang Chen, Yang Chen, Xueyin Chen, Shaonan Liu

**Affiliations:** 1 The Second Clinical College of Guangzhou University of Chinese Medicine, Guangzhou, China; 2 The Second Affiliated Hospital of Guangzhou University of Chinese Medicine (Guangdong Provincial Hospital of Chinese Medicine), Guangzhou, China

**Keywords:** digestive system cancers, gut microbiota, microbiota dysbiosis, tumorigenesis, tumor microenvironment

## Abstract

Digestive system cancers (DSCs) represent a substantial global health burden. In recent years, the role of gut microbiota in the DSCs has garnered considerable attention, but its change pattern during tumor progression and the specific mechanisms are still not fully understood. We conducted a comprehensive systematic review to characterize patterns of gut microbiota dysbiosis across different DSC types and assess their clinical significance. We systematically searched four English and three Chinese databases up to January 2025 to identify systematic reviews focused on the dynamic characteristics of the gut microbiota during gastrointestinal tumorigenesis. Microbiota biodiversity and taxonomic composition were extracted to identify specific signatures associated with DSCs. The ROBIS tool was used to evaluate the methodological quality of the included studies. Ultimately, 59 studies involving six distinct DSC types were included. Data synthesis and comparison revealed distinct microbiota profiles across DSCs. At the phylum level, Bacillota was decreased in esophageal cancer (EC) and pancreatic ductal adenocarcinoma (PDAC), Pseudomonadota was augmented in EC but exhibited divergent trajectories in colorectal cancer (CRC) and PDAC. Genus-level analyses revealed *Veillonella* enrichment in EC and PDAC, and *Fusobacterium* outgrowth in EC, gastric cancer (GC) and CRC. *Parvimonas* and *Streptococcus* showed a concordant ascending trend in GC and CRC. *Prevotella* was overrepresented in EC and GC. This synthesis delineates a qualitative landscape of gut microbiota imbalances associated with various DSCs, highlighting the potential for these microbial shifts to serve as markers for early detection and targeted therapy. Multiomics integration and prospective cohort studies should be prioritized to accelerate clinical translation.

## Introduction

Digestive system cancers (DSCs) are one of the most common and aggressive malignancies, accounting for 26% of global cancer incidence and 35% of cancer-related mortality. The burden of these diseases, along with their socioeconomic impact, continues to increase (Xie et al., 2021; Di et al., 2023; Danpanichkul et al., 2024). In recent years, DSCs have achieved significant advancements in endoscopic screening, imaging, pathological examination, and other diagnostic technologies. However, low diagnostic accuracy, primarily due to concealed early clinical symptoms of tumors, remains a critical obstacle (Shu et al., 2025; Zhan et al., 2025; Nijjar et al., 2024). Furthermore, despite the emergence of novel molecular targeted and immune therapies, drug resistance mechanisms, and toxic side effects continue to limit efficacy, resulting in unsatisfactory 5-year survival rates (Li et al., 2020; Chong et al., 2024). Therefore, elucidating the mechanisms behind tumorigenesis is paramount to identify early screening markers and develop innovative treatment strategies.

The gut microbiota, a diverse and complex microbial community colonizing in the intestine, profoundly regulates essential host functions—including intestinal homeostasis, immunomodulation and metabolic homeostasis—through intricate host-microbe interactions (Fan et al., 2023; Park et al., 2023). In recent years, accumulating evidence has highlighted the strong correlation between gut microbiota and tumor development, suggesting that microbial dysbiosis may contribute to the complexity of tumor initiation, progression, and prognosis (Zhang et al., 2022; Galeano Niño et al., 2022). Studies have found that patients with digestive tract tumors often exhibit characteristic, structural dysbiosis of the gut microbiota, specifically manifested as enrichment of proinflammatory pathobionts within the *Clostridium* and *Bacteroides* genera, and a concomitant depletion of protective commensal taxa such as *Bifidobacterium* (Chen et al., 2023; Wizenty and Sigal, 2025; Turocy and Crawford, 2025). Therefore, specific microbial signatures offer substantial potential as non-invasive biomarkers for the early detection of DSCs. Furthermore, microbial characteristics may facilitate the monitoring of tumor progression dynamics and the stratification of patient survival (Piccinno et al., 2025). These findings underscore the clinical utility and translational potential of the gut microbiota as a robust platform for the early detection and comprehensive management of DSCs.

Although numerous studies investigated the complex associations between gut microbiota and gastrointestinal tumors, existing studies still present significant fragmentation and heterogeneity. Marked discrepancies in clinicodemographic factors (e.g., region, diet, and tumor stage), alongside variations in sample sources (fecal vs. mucosal-associated microbiota), sequencing techniques, and analytical methods pipelines hinder the reproducibility of findings. Therefore, a systematic synthesis of existing evidence is warranted to clarify the common microbial signatures and clinical associations observed across DSCs. This summary aims to identify the common patterns in microbiota- tumor associations, reveal conflicting points in existing conclusions, and deeply analyze methodological challenges and to delineate the directions that future research should prioritize. Through a rigorous synthesis of evidence, this review expects to provide a theoretical basis for assessing the clinical utility of the gut microbiota in the diagnosis and management of DSCs.

## Methodology

### Data sources and searches

We conducted a comprehensive search of PubMed, Embase, Web of Science, the Cochrane Library, as well as China National Knowledge Infrastructure (CNKI), China Biology Medicine disc (CBM), and Wanfang databases, to retrieve all systematic reviews and meta-analyses examining the relationship between the gut microbiota and digestive-system tumorigenesis from database inception through January 9, 2025, with no language restrictions. We used Medical Subject Headings and free-text terms, including spelling variants and synonyms (See Supplementary Table 1 for the full retrieval strategy). Manually searched the reference lists of some systematic reviews to supplement the relevant studies.

### Eligibility criteria and study selection

Studies were included if they met all the following criteria: (1) systematic reviews or meta-analysis of observational studies; (2) patients diagnosed with digestive system malignancies; and (3) reporting on gut microbial diversity, taxonomic abundance, or specific microbiota shifts, or studies assessing correlations, and diagnostic accuracy measures. Studies without available full-text were excluded.

All search results were imported into reference management software (EndNote X9) to remove duplicates. Two researchers (WJ and LYK) independently screened titles and abstracts based on the inclusion criteria to exclude irrelevant articles, then assessed full-texts to select eligible studies. Any discrepancies were resolved by consulting a third experienced researcher (LSN).

### Data extraction

Data were extracted independently by two researchers (WJ, LYK) and subsequently reviewed by a third researcher (YY). Any disagreements were resolved through discussion with other team members (LSN, CXY and CY) as necessary. A Microsoft Excel spreadsheet, designed by the research team, was used to extract data. Researchers extracted the following data: study title; authors (year); types and number of the original studies included; sample size; tumor type; microbiota detection method; sample type and quality assessment tools. Pooled effect estimates (e.g., OR, AUC) with 95% confidence intervals (CI), measures of heterogeneity, and publication bias assessments were also extracted. Subgroup analysis results were also extracted if available.

### Methodological quality assessment

This study used the ROBIS tool (Whiting et al., 2018) to assess the risk of bias of included studies. ROBIS tool was divided into three phases: (1) assessing relevance (optional), (2) identifying concerns with the review process and (3) judging the overall risk of bias in the review. It includes five domains: (1) trial eligibility criteria; (2) identification and selection of trials; (3) data collection and trial appraisal; (4) synthesis and findings; and (5) interpretation of review findings. For each domain, researchers addressed the ROBIS signaling questions and judged the level of concern regarding bias (low, high, or no information) according to the ROBIS guidelines.

### Data analysis

Data on gut microbiota changes and digestive system tumors were extracted and analyzed. If the included studies had sufficient clinical and statistical homogeneity (defined as *I*^2^ < 50%), quantitative analysis was planned to use in R software (version 4.4.3) to pool the effect estimates with 95% CI; if the included studies were significantly heterogeneous (*I*^2^>50%), descriptive synthesis was intended to perform. Microbiota biodiversity metrics were scheduled to be represented by radar maps and differential microbiota patterns in varies of tumors were scheduled to be represented by Venn diagrams.

## Results

**Figure 1:**
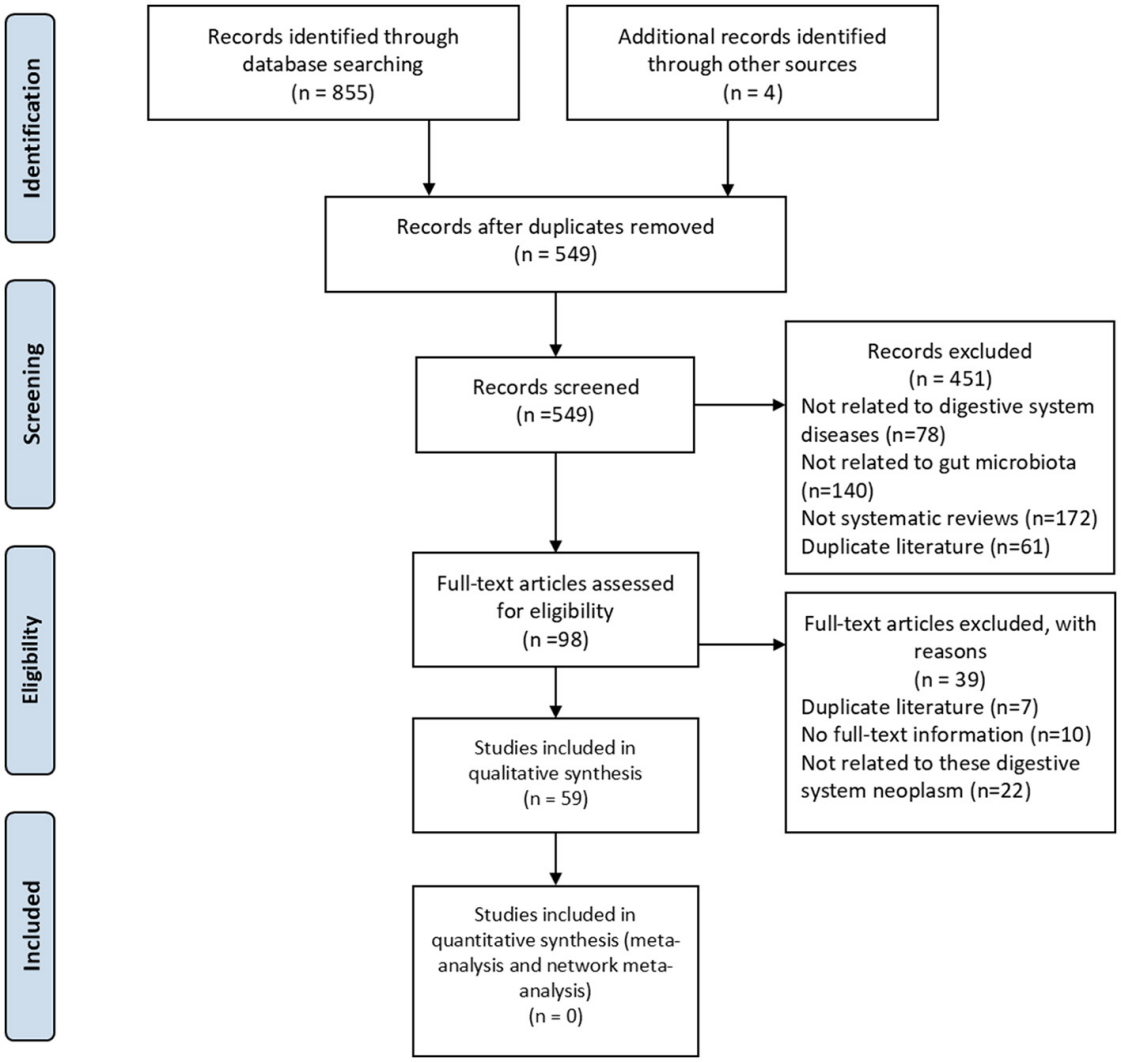
Flow diagram of study identification and selection.

A total of 859 publications investigating the relationship between the gut microbiota and DSCs were obtained through a systematic literature search. After removing duplicates, 549 publications were screened manually. Finally, 59 publications (52 in English and 7 in Chinese) published between 2001 to 2024 met the inclusion criteria and were included in the study (Supplementary Table 2).The process of literature searching and selecting was shown in Figure 1.

### Characteristics of included studies

The included studies covered six DSCs: colorectal cancer (CRC) (*n* = 37), gastric cancer (GC) (*n* = 11), pancreatic ductal adenocarcinoma (PDAC) (*n* = 6), esophageal cancer (EC) (*n* = 3), liver cancer (*n* = 1) and gallbladder cancer (*n* = 1). These studies primarily consisted of systematic reviews and meta-analyses based on cross-sectional, cohort, and case-control designs. The number of original studies included in the systematic reviews and meta-analyses ranged from 4 to 124. In addition, a total of 43 studies reported sample sizes, which varied considerably. Specifically, two studies reported sample sizes between 100 and 500, 15 studies reported sample sizes between 500 and 1,000, and 26 studies reported sample sizes greater than 1,000. Microbiota assays were reported in 48 studies: 15 employed 16S rRNA gene sequencing exclusively, five used shotgun metagenomic sequencing, three conducted whole-genome sequencing, and 25 utilized multiple complementary methodologies. Fifty studies reported sample types, with 47 of these involving fecal samples. Furthermore, mucosal, tissue, body fluid, and serum samples were also utilized in the included studies. Methodological quality was evaluated in 30 studies using 13 distinct instruments, including the Newcastle-Ottawa Scale (NOS). Given the significant methodological and clinical heterogeneity among the systematic reviews, and limited access to raw microbiota-abundance, we used qualitative pooling to analyze patterns of gut microbiota changes across DSC subtypes. The details were presented in Table 1.

**Table 1:** Characteristics of included studies.

Author (year)	Num.	Sample size	Tumor	Microbiota detection method	Sample type	Quality evaluation tool	Groups	Tumor stage
Deng et al. (2024)	10	*n* = 527	EC	16S rRNA	Stool, tumor/non-tumor mucosa samples	NOS	EC & HC	NM
Zhang et al. (2024)	9	*n* = 568	EC	16S rRNA	Fecal samples	NOS	EC & HC	NM
Vadhwana et al. (2023)	87	*n* = 8,831	EC	Culture, T-RFLP, 16S rRNA, RT-PCR, qPCR;	Tissue, fecal, saliva, tongue coating, plaque, gastric/esophageal fluid, serum, urine, mouthwash samples	NOS & STORMS	Cancer, benign & healthy	NM
(Chen et al. 2023)	16	*n* = 2,644	GC	16S rRNA	Gastric mucosa/swab, tongue coating, oral swab, stool, gastric fluid	Fast QC	GC & NGC	NM
Li et al. (2023)	11^*^	*n* = 2,198	GC	16S rRNA	Mucosa, fluid samples	NA	HC, gastritis, IM & GC	NM
Liu et al. (2022)	6	*n* = 825	GC	16S rRNA	Gastric biopsy samples	NA	SG, AG, IM & GC	NM
Wang et al. (2024)	11	*n* = 2,036	GC	16S rRNA	Gastric biopsy, stool samples	NA	GC, benign & HC	NM
Xue et al. (2001)	21^▴^	*n* = 9,747	GC	NA	NA	NA	GC, PLS & GAL	NM
(Yang et al. 2023)	9	*n* = 754	GC	16S rRNA	Fecal samples	NOS	GC & NGC	NM
Yang et al. (2021)	14^▴^	*n* = 1,543	GC	16S rRNA, 454 Pyrosequencing;	Endoscopic biopsy, surgical biopsy samples	NOS	GC & NGC	NM
Hu et al. (2006)	36	*n* = 7	GC	NA	NA	NA	GC & controls	Early GC
Liu et al. (2006)	25	*n* = 3,309,709	GC	NA	NA	NA	GC & controls	NM
Tian et al. (2006)	14	*n* = 3,811	GC	NA	NA	NA	GC & controls	NM
Xu et al. (2006)	10^▴^	*n* = 1,672	GC	NA	NA	NA	GC & controls	NM
Aidid et al. (2023)	7	*n* = 4,816	CRC	Culturing technique^1^, molecular & biochemical techniques^2^	Stool, colonic fluid, colonic tissue, Blood samples	Authors' judgement	CRC & control	1 study mentioned
Alhhazmi et al. (2023)	24^▴^	NA	CRC	16S rRNA, WGS, RT- PCR	Fecal, rectal mucosal biopsy, serum, Plasma specimen	PRISMA & STROBE	ADA, CRC & HC	9 studies mentioned
Amitay et al. (2018)	19	NA	CRC	qPCR, 16S rRNA	Fecal samples	NOS	CRC, ADA & HC	NM
Aprile et al. (2021)	19	NA	CRC	qPCR, MGS, 16S rRNA, ENTERO-test 24 plus + MALDI-TOF MS	Fecal, intestinal mucosa samples	NA	NA	NM
Avuthu et al. (2022)	5^*^	NA	CRC	SMS	Fecal samples	NA	CRC & HC	NM
Borges et al. (2015)	31	NA	CRC	FISH, qPCR, RT-qPCR, RISA, CS, CRA + AA, bft-typing, DNA extraction,	fecal, biopsy, mucosa samples	NA	NA	NM
Casimiro et al. (2022)	7^*^	*n* = 1,042	CRC	WGS	Fecal samples	NA	NA	NM
Costa et al. (2022)	39	*n* = 2,179	CRC	16S rRNA	Fresh/frozen tissue samples	NOS	CRC & HC, non-cancer mucosal	8 studies mentioned
Dai et al. (2018)	4^*^	*n* = 526	CRC	16S rRNA	Fecal samples	NA	CRC & controls	Early/late
Drewes et al. (2017)	3^*^	*n* = 1,301	CRC	16S rRNA, BF quantification	Stool, tumor tissue samples	NA	NA	mentioned
Eastmond et al. (2022)	13	*n* = 3,012	CRC	NA	Fecal samples	Cochrane risk of bias tool, JBI, NOS, SANRA	NA	1 study mentioned
Gaab et al. (2023)	1^*^/11^▴^	NA	CRC	Real-time qPCR, PCR	Tissue, stool samples	NOS	CRC & Controls	NM
Gao et al. (2023)	7^*^	*n* = 749	CRA	WGS, qRT-PCR	Fecal samples	NA	CRA & HC	NM
Gethings et al. (2020)	45	NA	CRC	qRT-PCR, 16S rRNA, FISH	Tissue, fecal, dental samples	NOS	CRC, HC & CRP^ 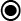 ^	NM
Gülhan et al. (2024)	9 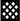	*n* = 1,579	CRC	whole genome shotgun, metagenome sequencing	Fecal samples	Fast QC	CRC, CRA & HC	NM
Herlo et al. (2024)	12	*n* = 2,883	CRC	16S rRN, RT-qPCR, MGS, DNA extraction	Stool, tissue, oral swabs, colonic mucosae	NA	CRC, CRA & HC	NM
Hussan et al. (2017)	90	NA	CRC	16S rRNA, qPCR, FISH, Whole genome shotgun, Metatranscriptomics	stool samples; tissue samples	NA	unidentified	evidence suggests
Kharofa et al. (2022)	11^ 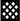 ^	*n* = 1,294	CRC	Fecal metagenomics sequencing	Fecal samples	NA	CRC, CRA & HC	NM
Liu et al. (2016)	6	*n* = 456	CRC	Fluorescent dye; qRT-PCR	Fecal samples	Original tool	CRC & HC	NM
Mo et al. (2020)	15	*n* = 2,099	CRC	16S rRNA	Stool, tissue samples (mucosa and biopsy specimens)	NA	CRC, CRA & HC	NM
Obón et al. (2022)	16^ 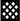 ^	*n* = 1,600	CRC	Shotgun sequencing	Fecal samples	NA	CRC, HC & P	NM
Ranjbar et al. (2021)	39	NA	CRC	qPCR, IFHA, MSM, qRT-PCR, ddPCR, IHC, FISH, 16S rRNA, ELISA	Tissue, fecal, saliva, serum, mucosa samples	NA	NA	NM
Riveros et al. (2023)	7^ 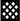 ^	*n* = 713	CRC	WGS	NA	Fast QC	CRC & Normal	NM
Shah et al. (2018)	9^ 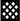 ^	*n* = 509	CRC	16S rRNA	Fecal samples	NA	CRC, CRA & Controls	NM
Sze et al. (2018)	14	*n* = 2,229	CRC	16S rRNA	Fecal, colon tissue samples	NA	CRC, CRA & Normal	NM
Tabowei et al. (2022)	9	NA	CRC	16S rRNA, PCR, qPCR	Stool, mucosa samples	NOS, SANRA, PRISMA	CRC, CRA & HC	NM
Thomas et al. (2019)	5^ 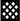 ^/2^*^	*n* = 969	CRC	Shotgun metagenomic	Fecal samples	NA	CRC, CRA & Controls	NM
Villar et al. (2022)	57	NA	CRC	PCR, FISH, MGS, ELISA, IF, GS, WB, 16S rRNA, CE, Serology, FIT, MTX,	Adjacent non-tumor tissue, tissue from tumor-free subjects, fecal, blood, saliva from cancer-free controls	An internal quality system	CRC, CRA & HC	NM
Wirbel et al. (2019)	8^ 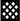 ^	*n* = 778	CRC	shotgun metagenomic	Fecal samples	NA	cancer & tumor-free controls	NM
Yu et al. (2022)	45	NA	CRC	NA	Fecal, oral, serum samples	NOS	NA	NM
Zwezerijnen et al. (2023)	28	NA	CRC	16S rRNA, qPCR, metagenomic shot-gun sequencing	Fecal, oral mucosa, serum samples	QUADAS-2	CRC & HC	Excluding late CRC
Jiang et al. (2021)	13^ 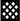 ^	*n* = 1,395	CRC	NA	Fecal samples	NA	CRC, UC, CD & Controls	NM
Wu et al. (2024)	11	*n* = 1,969	CRC	16S rRNA	Stool samples	NOS	CRC, AD & HC	NM
Dong et al. (2015)	23^▴^	*n* = 182,561	CRC	14C-UBT, IgG, CagA, Urease test Histopathology	NA	NA	CRC & Controls	NM
Zong et al. (2018)	15	NA	CRC	NA	Fecal samples	A standard	CRC & HC	NM
Islam et al. (2022)	82^▴^	*n* = 10,165	cancer	16S rRNA sequencing, shotgun sequencing	NA	NOS	Cases & Controls	NM
Vorstenbosch et al. (2023)	124	*n* = 15,764	cancer	NA	EC: unidentified; CRC: fecal, mucosal samples	NOS	Cases & Controls	NM
Trivedi et al. (2023)	17	NA	HCC	NA	NA	AMSTAR 2, SANRA, AXIS, NOS	NA	NM
Lederer et al. (2023)	12	*n* = 550	CCA	Cultivation methods; 16S rRNA	Fecal samples	NA	NA	NM
Mattos et al. (2022)	11^▴^/2/2^*^	*n* = 2,594	PDAC	16S rRNA, BD Phoenix system or Vitek-2 System	Saliva, stool, bile, duodenum, duodenal fluid	NHLBI, CBRG	NA	NM
Merali et al. (2024)	28^▴^/10 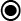	NA	PDAC	16S rRNA	blood plasma, biofluids, fresh tissue, FFPE pancreatic/oral/fecal samples	AMSTAR	NA	NM
Hong et al. (2024)	4^*^/8^▴^/2^ 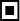 ^	*n* = 1,276	PDAC	16s rRNA gen, 18s rRNA, metagenomic sequencing	Duodenal fluid, stool samples	NOS	PDAC, CP & Controls	NM
Jankowski et al. (2024)	14	NA	PDAC	16S, 18S rRNA, MGS, WGS	Fecal, duodenal fluid	NA	PDAC & HC	NM
Memba et al. (2017)	9	NA	PDAC	16s rRNA	Fecal, saliva, blood samples	NOS	PDAC, CP & HC	NM
Xie et al. (2023)	7^ 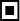 ^	*n* = 416	PDAC	16SrRNA, MGS	Fecal samples	AHRQ	PDAC & HC	NM

EC, esophageal cancer; NOS, Newcastle-Ottawa scale; HC, healthy control; NM, not mentioned; RT-PCR, reverse transcription polymerase chain reaction; STORMS, strengthening the organization and reporting of microbiome studies; GC, gastric cancer; NGC, non-gastric cancer; IM, intestinal metaplasia; SG, superficial gastritis; AG, atrophic gastritis; NA, not applicable; PLS, precancerous lesions of stomach; GAL, gastric lymphoma; CRC, colorectal cancer; WGS, whole genome sequencing; PRISMA, preferred reporting items for systematic reviews and meta-analyses; STROBE, strengthening the reporting of observational studies in epidemiology; ADA, advanced adenoma; MGS, metagenomic sequencing; SMS, shotgun metagenomic sequencing; FISH, fluorescence *in situ* hybridization; RISA, ribosomal intergenic spaur analysis; CS, clone sequencing; JBI, Joanna Briggs institute; SANRA, scale for the assessment of narrative review articles; CRA, colorectal adenoma; qRT-PCR/qPCR, quantitative real-time PCR; CRP, colorectal polyp; P, patients; ddPCR, droplet digital PCR; IHC, immunohistochemistry; ELISA, enzyme-linked immunosorbent assay; IF, immunofluorescence; WB, western blot; CE, capillary electrophoresis; FIT, fecal immunochemical test; QUADAS-2, quality assessment of diagnostic accuracy studies-2; UC, ulcerative colitis; CD, Crohn's disease; AD, adenoma; HCC, hepatocellular carcinoma; CCA, cholangiocarcinoma; AXIS, assessment of execution of intervention studies; PDAC, pancreatic ductal adenocarcinoma; CBRG, cochrane back review group; AMSTAR, a measurement tool to assess systematic reviews;^*^, Cohort; ▴, case-report; 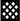, datasets; •, case-control; 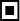, cross sectional study; 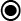, cancer.

### Microbial biodiversity

Twenty-four studies reported on α-diversity metrics of the gut microbiota, and eight also assessed β-diversity metrics. For α-diversity, 24 studies assessed six metrics, including Shannon index, Simpson index, Chao 1 index, Abundance-based Coverage Estimator (ACE), Operational Taxonomic Units (OTUs) and sobs (Supplementary Figure 1). The variation in α-diversity indices was inconsistent among case groups. In terms of β-diversity, the results were considered significantly different. Details of these metrics are provided in Supplementary Table 4.

### Alterations in gut microbiota composition

**Figure 2:**
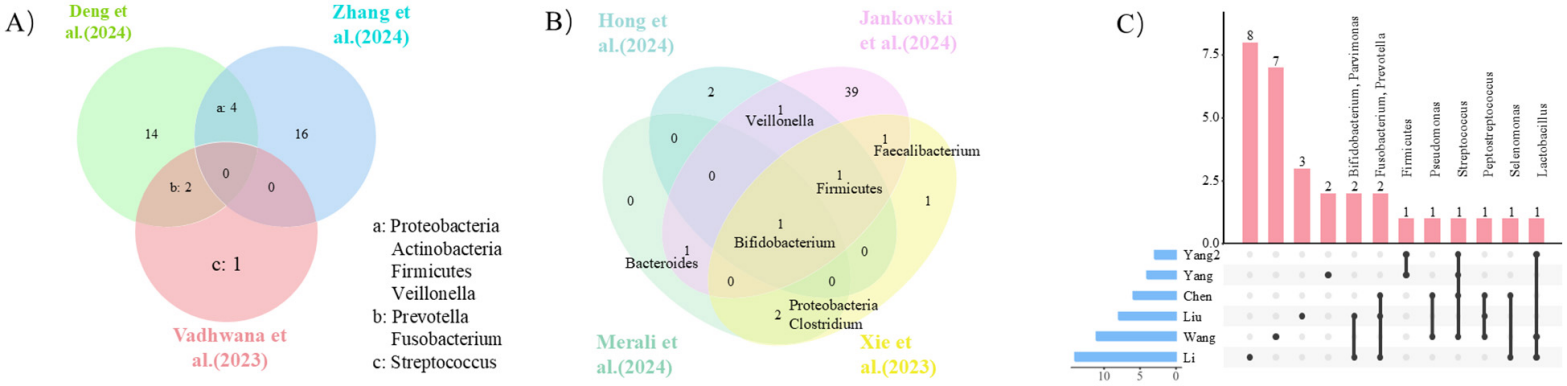
Alterations in gut microbiota composition in DSCs. **(A)**: Esophageal cancer; **(B)**: Pancreatic ductal adenocarcinoma; **(C)**: Gastric cancer.

**Figure 3:**
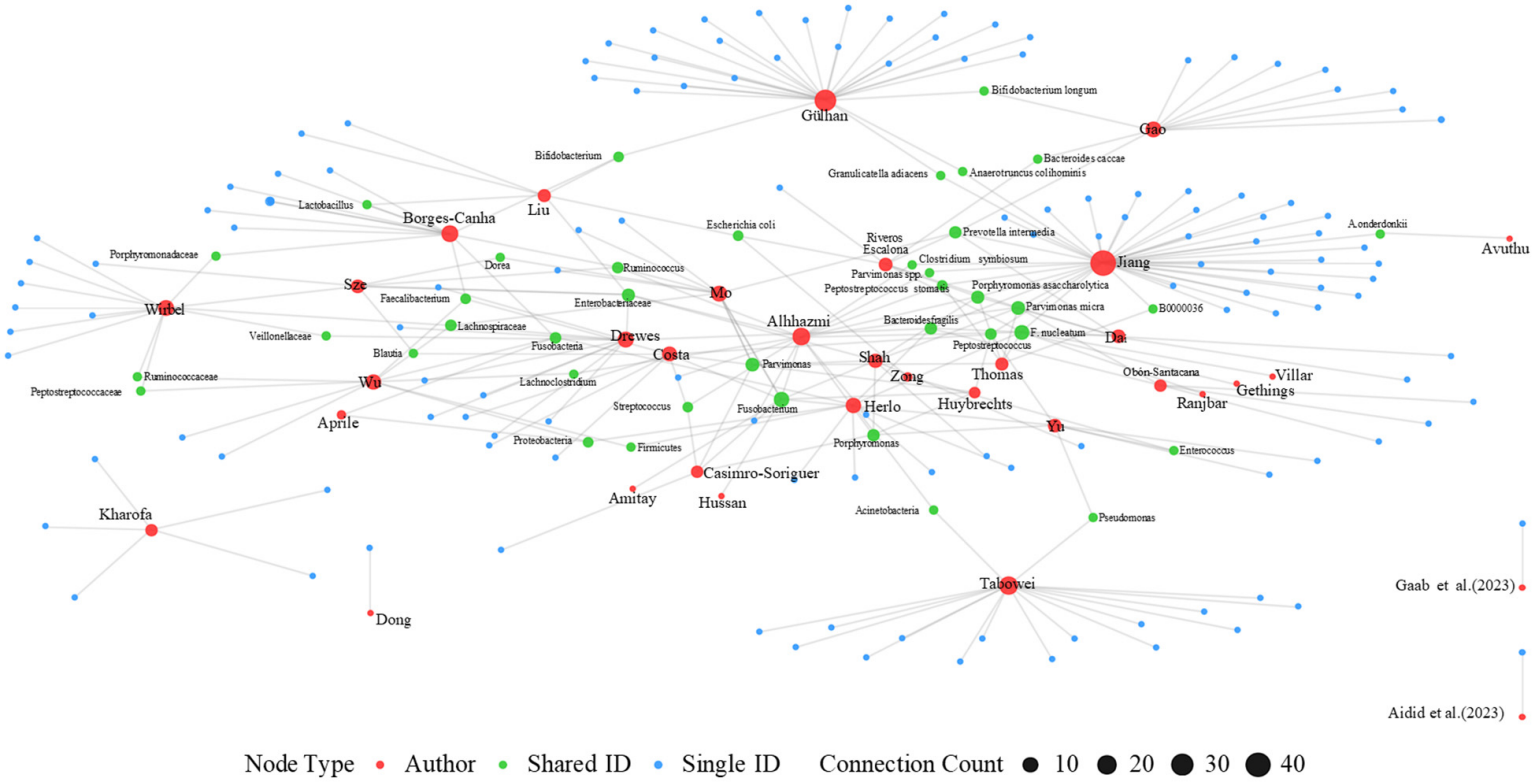
Alterations in gut microbiota composition in colorectal cancer.

Forty-eight studies reported alterations in the gut microbiota of DSC patients (Figures 2, 3). The Taxonomy Browser, developed by the National Center for Biotechnology Information (NCBI), was utilized to summarize changes in microbiota at various taxonomic levels between the case groups and control groups (Supplementary Table 4).

At the phylum level, compared with the control group, patients with EC showed increased abundance of Pseudomonadota (formerly known as Proteobacteria) and a decreased abundance of Bacillota (formerly known as Firmicutes); changes in Actinomycetota (formerly known as Actinobacteria) was inconsistent across studies. In the GC group, findings on the abundance of Bacillota were also contradictory. There were differences in the abundance of Pseudomonadota and Clostridia between CRC and the control group. In the PDAC group, the abundance of Bacillota decreased and changes of Pseudomonadota were not parallel. At the family level, the abundance of Enterobacteriaceae increased in the CRC group, while shifts in the abundance of Lachnospiraceae, Oscillospiraceae (formerly known as Ruminococcaceae) and Peptostreptococcaceae were variable. At the genus level, the abundance of *Prevotella, Veillonella* and *Fusobacterium* was higher in the EC patients than in the controls. In the GC group, the abundance of *Streptococcus, Fusobacterium, Prevotella, Parvimonas* and *Lactobacillus* increased, while findings for *Pseudomonas, Selenomonas* and *Bifidobacterium* were inconsistent. In the CRC group, the levels of *Fusobacterium, Parvimonas* and *Streptococcus* were higher, the abundance of *Ruminococcus* was variable, and *Porphyromonas* was pointed out to be associated with CRC. In the PDAC group, the abundance of *Bacteroides* and *Veillonella* was higher, while that of *Faecalibacterium* was lower, and abundance shifts for *Bifidobacterium* and *Clostridium* were contradictory. At the species level, compared to controls, *Fusobacterium nucleatum* (*F. nucleatum*), *Parvimonas micra, Bacteroides fragilis, Prevotella intermedia*, and *Clostridium symbiosum* were higher in the CRC group. Furthermore, *Porphyromonas asaccharolytica* was associated with CRC, while *Peptostreptococcus stomatis* was considered as a potential disease biomarker. Additionally, only two studies provided preliminary evidence regarding liver cancer and biliary tract cancers, indicating an enrichment of pathogenic bacteria alongside a reduced abundance of beneficial bacteria in patients with liver cancer.

### Correlation and diagnostic accuracy indicators

Eleven of the included studies explored the association between the gut microbiota and gastrointestinal tumors, primarily utilizing the odds ratio (OR) as the measure of association. In addition, 7 articles reported diagnostic accuracy indicators for different diagnostic markers. Among the studies exploring the association, 5 studies investigated the link between *Helicobacter pylori* (*H. pylori*) and GC (OR range: 2.00–4.16), indicating that *H. pylori* infection increased the risk of GC. The remaining 6 studies assessed the associations with CRC, identifying positive correlations with polyketide synthase (pks+) *Escherichia coli* (OR 2.27; 95% CI: 1.13–4.57), *F. nucleatum* (OR range: 2.42–4.558), and *H. pylori* (OR 1.44; 95%CI: 1.39–1.48). More detailed data were shown in Supplementary Table 4.

### Methodological quality assessment

**Figure 4:**
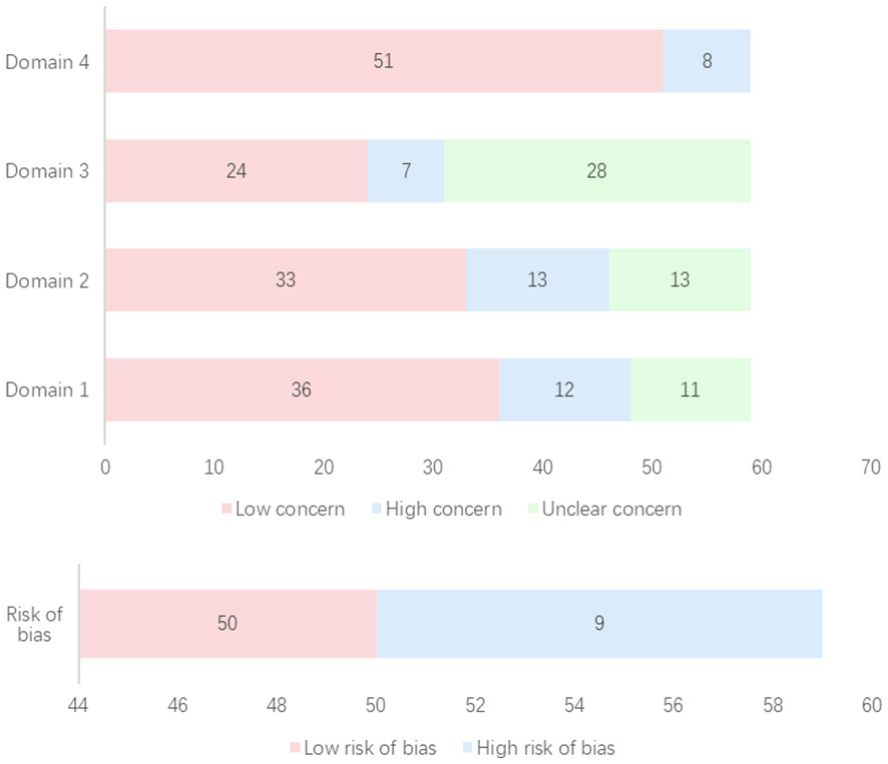
Results of methodological quality evaluation. Domain 1 Study eligibility criteria; Domain 2 Identification and selection of studies; Domain 3 Data collection and study appraisal; Domain 4 Synthesis and findings.

Methodological quality was appraised using the ROBIS tool across four domains. As summarized in Figure 4, most studies demonstrated a low concern for bias, while nine studies were identified as having a high risk. Comprehensive details of the assessment are provided in Supplementary Table 3.

## Discussion

In this study, we systematically synthesized the patterns of gut microbiota alterations in DSCs through a narrative synthesis of published systematic reviews and meta-analyses. For several malignancies, we observed substantial differences in the composition of gut microbiota between the case and control groups, indicating potentially significant associations between microbial dysbiosis and tumorigenesis. Specifically, we found that although reports on α-diversity were inconsistent across studies, likely reflecting differences in sample types, sequencing methods, data preprocessing protocols, and inter-individual variability, β-diversity demonstrated significant differences across several DSC types. Secondly, descriptive pooled results showed that, at the phylum level, Bacillota abundance was decreased in EC and PDAC patients. Although Pseudomonadota levels were increased in EC, they exhibited discordant trends in CRC and PDAC. At the genus level, *Veillonella* was elevated in EC and PDAC groups, whereas the abundance of *Fusobacterium* was higher in EC, GC, and CRC relative to matched controls. *Parvimonas* and *Streptococcus* showed a consistent upward trend in gastric and colorectal malignancies. *Prevotella* showed overexpression in EC and GC, while *Bifidobacterium* showed inconsistent trends in GC and PDAC groups. These findings are consistent with the results of current studies. Changes in microbial biodiversity and characteristics are merely phenotypic manifestations of microorganism-tumor interactions. This review deeply analyzes the dynamic interaction network of microbial metabolic reprogramming, immune regulation and tumor-microenvironment remodeling behind these changes.

### Cancer-promoting mechanism of changes in microbial biodiversity

Our study found significant changes in bacterial diversity (especially β-diversity) in tumor patients, and we explored the role of diversity changes in tumor development and progression and their clinical significance. Abnormal changes in gut microbiota diversity are closely related to microbiota imbalance (García Menéndez et al., 2024). Microbiota imbalance potentially affects tumor progression through multiple mechanisms, which displayed in Supplementary Figure 2. Specifically, microbial flora imbalance often alters the composition and abundance of microbial metabolites. These metabolic shifts can increase intestinal permeability, ultimately facilitating the translocation of pathogenic bacteria, such as *Escherichia coli* (*E. coli*), and their harmful byproducts into the bloodstream (Sun et al., 2024). Microbial dysbiosis is also closely linked to the remodeling of tumor microenvironment (TME) (Galeano Niño et al., 2022; Sepich-Poore et al., 2021; Nejman et al., 2020), potentially affecting metabolic reprogramming, immune evasion, and the vascular niche of tumor cells (Ganapathy-Kanniappan, 2018; Yang et al., 2023).

By changing the acid-base balance and oxygen concentration within the TME, abnormally activating the immune system, regulating the expression of immune checkpoint molecules, promoting angiogenesis and providing nutritional and energy support for tumor cells, gut microbes may actively promote tumor growth and spread (Xie et al., 2024; Liu et al., 2024; Deng et al., 2025). In addition, shifts in gut microbiota composition can influence the efficacy of immune checkpoint blockade (ICB), thereby facilitating immune evasion and tumor progression (Zhu et al., 2025; Huang et al., 2025). Therefore, monitoring changes in microbial diversity can provide diagnostic insights for clinicians and serve as a non-invasive biomarker for the early detection of tumors (Zitvogel et al., 2024).

### Determinants of taxonomic inconsistency across DSC cohorts

Our study identified a high degree of consistency in the trends of certain taxa; however, others—particularly at the species level—varied markedly across studies. This inconsistency can be systematically attributed to several layers of biological and methodological heterogeneity. First, host-related covariates, such as intestinal transit time and medication use, can substantially modulate the gut ecosystem, potentially masking primary disease-associated signals (Krukowski et al., 2026). For instance, taxa such as *Ruminococcus*, which exhibit divergent trajectories across DSC cohorts, have been shown to be highly sensitive to these external and internal variables. This complexity is further compounded by individual genetic backgrounds and immune status (Chai et al., 2023; Parizadeh and Arrieta, 2023). In addition to host factors, the choice of sample type can also introduce significant variation. Studies have shown that mucosal samples more accurately reflect the host–microbe interface than fecal samples (Ahn et al., 2025). Different intestinal regions and the contrast between tumor and adjacent tissues present distinct microenvironments—characterized by pH gradients, nutrient availability, and oxygen tension—which determine the local distribution of genera such as *Enterococcus, Bacteroides*, and *Clostridium* (Yu et al., 2025; Yu and Yang, 2020). These biological differences are further confounded by differences in profiling methods. Studies found significant differences between species-level relative-abundance estimates based on 16S rRNA amplicon and shotgun metagenomic sequencing results. This is mainly because 16S rRNA sequencing is influenced by primer preference and variation in 16S rRNA gene copy number, whereas metagenomic sequencing avoids amplification bias and offers greater taxonomic resolution and more realistically reflects the microbial genomic content and biomass (Strokach et al., 2025). Furthermore, geographic and dietary backgrounds critically shape baseline microbiota composition and individual susceptibility, while tumor-stage variations contribute an additional layer of complexity to the microbial landscape (Wong and Yu, 2023).

### Mechanism of specific microbiota

Nevertheless, our analysis detected highly consistent genus-level changes for several taxa, notably *Streptococcus, Prevotella, Veillonella, Fusobacterium*, and *Parvimonas*. It is worth noting that these five genera are typical oral taxa, suggesting a potential role for oral microbiota in the development of digestive-tract tumors. *Streptococcus*, as an opportunistic pathobiont, is widely present in the human gastrointestinal tract and nasopharynx. Numerous studies have reported its increased abundance in various gastrointestinal diseases, and its pathogenic potential is widely recognized (Yu et al., 2021). Certain strains of *Streptococcus* can inhibit the differentiation and infiltration of CD8^+^ T cells, activate the arginine metabolic pathway, promote lactate accumulation, and thereby remodel the tumor immune microenvironment to weaken antitumor immunity and support tumor growth and survival (Yuan et al., 2024). *Prevotella* is a genus of Gram-negative anaerobes that colonizes various mucosal sites, notably the oral cavity and gastrointestinal tract. It may drive inflammation and carcinogenesis by producing lipopolysaccharides (LPS), which activate oncogenic signaling cascades such as the IL-6/JAK1/STAT3 pathway. Interestingly, Zhang et al. observed that *Prevotella* was enriched in GC tissue; however, depleted in saliva (Zhang et al., 2021; Huang et al., 2021). This suggests that ectopic colonization of the gastrointestinal tract by oral-derived *Prevotella* may contribute to tumorigenesis. *Veillonella*, a common Gram-negative anaerobic coccus distributed in the oral cavity, pharynx, and the respiratory and intestinal tracts, can promote a tumor-supporting microenvironment through a triad of inflammation, metabolic reprogramming, and immune modulation. The LPS component of its cell wall activates the TLR4–NF-κB signaling cascade, inducing the secretion of pro-inflammatory cytokines and establishing a chronic inflammatory milieu that facilitates epithelial–mesenchymal transition (EMT). Moreover, its metabolic activities can reduce the local pH through organic acid production, thereby upregulating HIF-1α and VEGF expression to promote angiogenesis, and enhancing tumor invasiveness. In parallel, these metabolites elevate the expression of immune checkpoint molecules such as PD-L1 and CTLA-4, promote regulatory T cell (Treg) expansion, and suppress CD8^+^ T cell effector function, collectively contributing to tumor immune evasion (Yan et al., 2024; Shakhpazyan et al., 2024; Zhang et al., 2023). *Fusobacterium* has been detected in infected gastrointestinal tissues, likely reflecting selective translocation from the oral cavity. Some species (e.g., *F. nucleatum*) attach to and colonize the intestine through FadA and Fap2 proteins, favoring a proinflammatory microenvironment that promotes tumor growth (Situ et al., 2025). *Parvimonas* has been associated with CRC. Studies report it promotes infiltration of Th2 and Th17 cells into tumor tissue, depletes Th1 cells, and modulates inflammatory responses in ways that facilitate tumor occurrence and development (Ternes et al., 2020; Lee et al., 2024). The mechanisms attributed to these genera are not isolated. They may affect tumor development in a synergistic or antagonistic manner (Tropini et al., 2017).

### Limitations and future directions

Limitations of this study include the following. First, the evidence for certain types of malignancies, particularly liver and biliary tract cancers, is sparse; only one systematic review was available for each. Most current studies did not adequately account for tumor; although we performed stratified analysis by tumor site, information regarding tumor stage and precise tumor site was insufficient. Large interstudy differences in sample type, sequencing technique, and geographic population precluded quantitative pooling. In addition, we could not fully distinguish the distinct effects of intestinal vs. intratumoral microbiota on tumor progression. Numerous host, environmental, and technical factors influence microbiota composition, making it difficult to accurately analyze its impact on tumors. Finally, current research has not clarified the causal relationship between gut microbiota and tumors.

This umbrella review highlights a complex role of gut microbiota in tumor progression and highlights its importance in early cancer screening. In the future, in-depth research on underrepresented DSC types (such as liver cancer and gallbladder cancer) might provide the necessary clinical evidence to refine diagnostic and prognostic approaches. To generate high-quality quantitative evidence, future studies should adopt standardized protocols and incorporate multiomics techniques to explore the functional profiles of intestinal microbes and further analyze their distinct roles across different tumor stages. Analysis of real-world data using correlation tools such as variable analysis and Mendelian randomization to mine possible causal relationships between gut microbiota and tumors is also a feasible direction for future research. Continuing to focus on developing markers for early tumor diagnosis, and combining AI-assisted diagnostic tools to improve diagnostic accuracy and efficiency are important challenges going forward (Marra et al., 2025). Lastly, animal experiments and *in vitro* cell assays are necessary to develop and test interventions aimed at counteracting tumor progression by manipulating the microbial flora.

## Conclusion

In this study, we mapped the gut microbiota imbalances associated with the most prevalent DSCs. Our analysis suggests *Veillonella* as a potential marker for PDAC, Pseudomonadota as a potential marker for EC, and selective enrichment of *Clostridium, Parvimonas*, and *Streptococcus* across several DSCs, with the exception of PDAC. Although our findings provide a foundational framework for understanding microbiota alterations in DSCs, the limited evidence for liver and biliary tract malignancies necessitates large-scale, geographically diverse validation studies to confirm their specific microbial signatures.

## Data Availability

The original contributions presented in the study are included in the article/Supplementary material, further inquiries can be directed to the corresponding authors.
