# Supplementary material for: Characterization of dysbiosis patterns in gut microbiota of digestive system cancers: an umbrella review
Source: Front Microbiol. 2026 Apr 28;17:1782471. doi: 10.3389/fmicb.2026.1782471 (PMC13161176; doi:10.3389/fmicb.2026.1782471)
Supplement: Supplementary file 1 [file Table_1.docx]

**Table S1 Detailed search strategy**

**PubMed: January 9, 2025**

**Search Strategy:**

| Search number | Search Details |
| --- | --- |
| 1 | "Digestive System Neoplasms"[MeSH Terms] |
| 2 | "Digestive System Neoplasms"[Title/Abstract] OR "Digestive System tumor"[Title/Abstract] OR "Digestive System cancer"[Title/Abstract] |
| 3 | "Gastrointestinal Neoplasms"[MeSH Terms] |
| 4 | "Gastrointestinal Neoplasms"[Title/Abstract] OR "Gastrointestinal tumor"[Title/Abstract] OR "Gastrointestinal cancer"[Title/Abstract] |
| 5 | "Esophageal Neoplasms"[MeSH Terms] |
| 6 | "Esophageal Neoplasms"[Title/Abstract] OR "Esophageal cancer"[Title/Abstract] OR "Esophageal tumor"[Title/Abstract] |
| 7 | "Stomach Neoplasms"[MeSH Terms] |
| 8 | "Stomach Neoplasms"[Title/Abstract] OR "Stomach cancer"[Title/Abstract] OR "Stomach tumor"[Title/Abstract] OR "gastric neoplasms"[Title/Abstract] OR "gastric cancer"[Title/Abstract] OR "gastric tumor"[Title/Abstract] |
| 9 | "Duodenal Neoplasms"[MeSH Terms] |
| 10 | "Duodenal Neoplasms"[Title/Abstract] OR "Duodenal cancer"[Title/Abstract] OR "Duodenal tumor"[Title/Abstract] OR "small bowel cancer"[Title/Abstract] OR "small bowel tumor"[Title/Abstract] OR "small intestine cancer"[Title/Abstract] OR "small intestine tumor"[Title/Abstract] |
| 11 | "Appendiceal Neoplasms"[MeSH Terms] |
| 12 | "Appendiceal Neoplasms"[Title/Abstract] OR "Appendiceal tumor"[Title/Abstract] OR "Appendiceal cancer"[Title/Abstract] |
| 13 | "Colonic Neoplasms"[MeSH Terms] |
| 14 | "Colonic Neoplasms"[Title/Abstract] OR "Colonic cancer"[Title/Abstract] OR "Colonic tumor"[Title/Abstract] |
| 15 | "Rectal Neoplasms"[MeSH Terms] |
| 16 | "Rectal Neoplasms"[Title/Abstract] OR "Rectal cancer"[Title/Abstract] OR "Rectal tumor"[Title/Abstract] |
| 17 | "Liver Neoplasms"[MeSH Terms] |
| 18 | "Liver Neoplasms"[Title/Abstract] OR "Liver cancer"[Title/Abstract] OR "Liver tumor"[Title/Abstract] |
| 19 | "Gallbladder Neoplasms"[MeSH Terms] |
| 20 | "Gallbladder Neoplasms"[Title/Abstract] OR "Gallbladder cancer"[Title/Abstract] OR "Gallbladder tumor"[Title/Abstract] |
| 21 | "Pancreatic Neoplasms"[MeSH Terms] |
| 22 | "Pancreatic Neoplasms"[Title/Abstract] OR "Pancreatic cancer"[Title/Abstract] OR "Pancreatic tumor"[Title/Abstract] |
| 23 | "Microbiota"[MeSH Terms] |
| 24 | "Gastrointestinal Microbiome"[MeSH Terms] |
| 25 | "Gastrointestinal Microbiome"[Title/Abstract] OR "Microbiota"[Title/Abstract] OR "flora"[Title/Abstract] |
| 26 | "overview"[Title] OR "umbrella review"[Title] OR "systematic review"[Title] OR "meta"[Title] |
| 27 | "Digestive System Neoplasms"[MeSH Terms] OR "Digestive System Neoplasms"[Title/Abstract] OR "Digestive System tumor"[Title/Abstract] OR "Digestive System cancer"[Title/Abstract] OR "Gastrointestinal Neoplasms"[MeSH Terms] OR "Gastrointestinal Neoplasms"[Title/Abstract] OR "Gastrointestinal tumor"[Title/Abstract] OR "Gastrointestinal cancer"[Title/Abstract] OR "Esophageal Neoplasms"[MeSH Terms] OR "Esophageal Neoplasms"[Title/Abstract] OR "Esophageal cancer"[Title/Abstract] OR "Esophageal tumor"[Title/Abstract] OR "Stomach Neoplasms"[MeSH Terms] OR "Stomach Neoplasms"[Title/Abstract] OR "Stomach cancer"[Title/Abstract] OR "Stomach tumor"[Title/Abstract] OR "gastric neoplasms"[Title/Abstract] OR "gastric cancer"[Title/Abstract] OR "gastric tumor"[Title/Abstract] OR "Duodenal Neoplasms"[MeSH Terms] OR "Duodenal Neoplasms"[Title/Abstract] OR "Duodenal cancer"[Title/Abstract] OR "Duodenal tumor"[Title/Abstract] OR "small bowel cancer"[Title/Abstract] OR "small bowel tumor"[Title/Abstract] OR "small intestine cancer"[Title/Abstract] OR "small intestine tumor"[Title/Abstract] OR "Appendiceal Neoplasms"[MeSH Terms] OR "Appendiceal Neoplasms"[Title/Abstract] OR "Appendiceal tumor"[Title/Abstract] OR "Appendiceal cancer"[Title/Abstract] OR "Colonic Neoplasms"[MeSH Terms] OR "Colonic Neoplasms"[Title/Abstract] OR "Colonic cancer"[Title/Abstract] OR "Colonic tumor"[Title/Abstract] OR "Rectal Neoplasms"[MeSH Terms] OR "Rectal Neoplasms"[Title/Abstract] OR "Rectal cancer"[Title/Abstract] OR "Rectal tumor"[Title/Abstract] OR "Liver Neoplasms"[MeSH Terms] OR "Liver Neoplasms"[Title/Abstract] OR "Liver cancer"[Title/Abstract] OR "Liver tumor"[Title/Abstract] OR "Gallbladder Neoplasms"[MeSH Terms] OR "Gallbladder Neoplasms"[Title/Abstract] OR "Gallbladder cancer"[Title/Abstract] OR "Gallbladder tumor"[Title/Abstract] OR "Pancreatic Neoplasms"[MeSH Terms] OR "Pancreatic Neoplasms"[Title/Abstract] OR "Pancreatic cancer"[Title/Abstract] OR "Pancreatic tumor"[Title/Abstract] |
| 28 | "Microbiota"[MeSH Terms] OR "Gastrointestinal Microbiome"[MeSH Terms] OR "Gastrointestinal Microbiome"[Title/Abstract] OR "Microbiota"[Title/Abstract] OR "flora"[Title/Abstract] |
| 29 | ("Digestive System Neoplasms"[MeSH Terms] OR ("Digestive System Neoplasms"[Title/Abstract] OR "Digestive System tumor"[Title/Abstract] OR "Digestive System cancer"[Title/Abstract]) OR "Gastrointestinal Neoplasms"[MeSH Terms] OR ("Gastrointestinal Neoplasms"[Title/Abstract] OR "Gastrointestinal tumor"[Title/Abstract] OR "Gastrointestinal cancer"[Title/Abstract]) OR "Esophageal Neoplasms"[MeSH Terms] OR ("Esophageal Neoplasms"[Title/Abstract] OR "Esophageal cancer"[Title/Abstract] OR "Esophageal tumor"[Title/Abstract]) OR "Stomach Neoplasms"[MeSH Terms] OR ("Stomach Neoplasms"[Title/Abstract] OR "Stomach cancer"[Title/Abstract] OR "Stomach tumor"[Title/Abstract] OR "gastric neoplasms"[Title/Abstract] OR "gastric cancer"[Title/Abstract] OR "gastric tumor"[Title/Abstract]) OR "Duodenal Neoplasms"[MeSH Terms] OR ("Duodenal Neoplasms"[Title/Abstract] OR "Duodenal cancer"[Title/Abstract] OR "Duodenal tumor"[Title/Abstract] OR "small bowel cancer"[Title/Abstract] OR "small bowel tumor"[Title/Abstract] OR "small intestine cancer"[Title/Abstract] OR "small intestine tumor"[Title/Abstract]) OR "Appendiceal Neoplasms"[MeSH Terms] OR ("Appendiceal Neoplasms"[Title/Abstract] OR "Appendiceal tumor"[Title/Abstract] OR "Appendiceal cancer"[Title/Abstract]) OR "Colonic Neoplasms"[MeSH Terms] OR ("Colonic Neoplasms"[Title/Abstract] OR "Colonic cancer"[Title/Abstract] OR "Colonic tumor"[Title/Abstract]) OR "Rectal Neoplasms"[MeSH Terms] OR ("Rectal Neoplasms"[Title/Abstract] OR "Rectal cancer"[Title/Abstract] OR "Rectal tumor"[Title/Abstract]) OR "Liver Neoplasms"[MeSH Terms] OR ("Liver Neoplasms"[Title/Abstract] OR "Liver cancer"[Title/Abstract] OR "Liver tumor"[Title/Abstract]) OR "Gallbladder Neoplasms"[MeSH Terms] OR ("Gallbladder Neoplasms"[Title/Abstract] OR "Gallbladder cancer"[Title/Abstract] OR "Gallbladder tumor"[Title/Abstract]) OR "Pancreatic Neoplasms"[MeSH Terms] OR ("Pancreatic Neoplasms"[Title/Abstract] OR "Pancreatic cancer"[Title/Abstract] OR "Pancreatic tumor"[Title/Abstract])) AND ("Microbiota"[MeSH Terms] OR "Gastrointestinal Microbiome"[MeSH Terms] OR ("Gastrointestinal Microbiome"[Title/Abstract] OR "Microbiota"[Title/Abstract] OR "flora"[Title/Abstract])) AND ("overview"[Title] OR "umbrella review"[Title] OR "systematic review"[Title] OR "meta"[Title]) |

**Web of Science Core Collection: January 9, 2025**

**Science Citation Index Expanded (SCI-EXPANDED) – from 1995 to now**

**Search Strategy:**

| # | Search Query |
| --- | --- |
| 1 | TS=("Digestive System Neoplasms" OR "Digestive System tumor" OR "Digestive System cancer" OR "Gastrointestinal Neoplasms" OR "Gastrointestinal tumor" OR "Gastrointestinal cancer" OR "Esophageal Neoplasms" OR "Esophageal cancer" OR "Esophageal tumor" OR "Stomach Neoplasms" OR "Stomach cancer" OR "Stomach tumor" OR "gastric neoplasms" OR "gastric cancer" OR "gastric tumor" OR "Duodenal Neoplasms" OR "Duodenal cancer" OR "Duodenal tumor" OR "small bowel cancer " OR "small bowel tumor" OR "small bowel neoplasms" OR "small intestine cancer " OR "small intestine tumor " OR "small intestine neoplasms " OR "Appendiceal Neoplasms" OR "Appendiceal tumor" OR "Appendiceal cancer" OR "Colonic Neoplasms" OR "Colonic cancer" OR "Colonic tumor" OR "Rectal Neoplasms" OR "Rectal cancer" OR "Rectal tumor" OR "Liver Neoplasms" OR "Liver cancer" OR "Liver tumor" OR "Gallbladder Neoplasms" OR "Gallbladder cancer" OR "Gallbladder tumor" OR "Pancreatic Neoplasms" OR "Pancreatic cancer" OR "Pancreatic tumor" ) Editions: WOS.SCI |
| 2 | TS=("Gastrointestinal Microbiome" OR Microbiota OR flora) Editions: WOS.SCI |
| 3 | TI=(overview OR "umbrella review" OR "systematic review" OR meta) Editions: WOS.SCI |
| 4 | #4 AND #3 AND #2 Editions: WOS.SCI |

**Embase: January 9, 2025**

**Search Strategy:**

| No. | Query Results |
| --- | --- |
| #1. | 'digestive system tumor'/exp |
| #2. | 'gastrointestinal tumor'/exp |
| #3. | 'esophagus tumor'/exp |
| #4. | 'stomach tumor'/exp |
| #5. | 'duodenum tumor'/exp |
| #6. | 'small intestine cancer'/exp |
| #7. | 'appendix tumor'/exp |
| #8. | 'colon tumor'/exp |
| #9. | 'rectum tumor'/exp |
| #10. | 'liver tumor'/exp |
| #12. | 'pancreas tumor'/exp |
| #13. | 'microflora'/exp |
| #14. | 'intestine flora'/exp |
| #15. | 'gastrointestinal microbiome':ab,ti OR microbiota:ab,ti OR flora:ab,ti |
| #16. | overview:ti OR 'umbrella review':ti OR 'systematic review':ti OR meta:ti |
| #17. | 'digestive system neoplasms':ab,ti OR 'digestive system tumor':ab,ti OR 'digestive system cancer':ab,ti OR 'gastrointestinal neoplasms':ab,ti OR 'gastrointestinal tumor':ab,ti OR 'gastrointestinal cancer':ab,ti OR 'esophageal neoplasms':ab,ti OR 'esophageal cancer':ab,ti OR 'esophageal tumor':ab,ti OR 'stomach neoplasms':ab,ti OR 'stomach cancer':ab,ti OR 'stomach tumor':ab,ti OR 'gastric neoplasms':ab,ti OR 'gastric cancer':ab,ti OR 'gastric tumor':ab,ti OR 'duodenal neoplasms':ab,ti OR 'duodenal cancer':ab,ti OR 'duodenal tumor':ab,ti OR 'small bowel cancer':ab,ti OR 'small bowel tumor':ab,ti OR 'small bowel neoplasms':ab,ti OR 'small intestine cancer':ab,ti OR 'small intestine tumor':ab,ti OR 'small intestine neoplasms':ab,ti OR 'appendiceal neoplasms':ab,ti OR 'appendiceal tumor':ab,ti OR 'appendiceal cancer':ab,ti OR 'colonic neoplasms':ab,ti OR 'colonic cancer':ab,ti OR 'colonic tumor':ab,ti OR 'rectal neoplasms':ab,ti OR 'rectal cancer':ab,ti OR 'rectal tumor':ab,ti OR 'liver neoplasms':ab,ti OR 'liver cancer':ab,ti OR 'liver tumor':ab,ti OR 'gallbladder neoplasms':ab,ti OR 'gallbladder cancer':ab,ti OR 'gallbladder tumor':ab,ti OR 'pancreatic neoplasms':ab,ti OR 'pancreatic cancer':ab,ti OR 'pancreatic tumor':ab,ti |
| #18. | #1 OR #2 OR #3 OR #4 OR #5 OR #6 OR #7 OR #8 OR #9 OR #10 OR #11 OR #12 OR #17 |
| #19. | #13 OR #14 OR #15 |
| #20. | #16 AND #18 AND #19 |

**The Cochrane Library: January 9, 2025**

**Search Strategy:**

| # | Search Query |
| --- | --- |
| #1 | ("Gastrointestinal Microbiome" OR Microbiota OR flora):ti.ab,kw |
| #2 | (overview OR "umbrella review" OR "systematic review" OR meta):ti,ab,kw |
| #3 | ("Diaestive Svstem Neoplasms" OR "Digestive System tumor" OR "DigestiveSvstem cancer" OR "Gastrointestinal Neoplasms" OR "Gastrointestinal tumor'OR "Gastrointestinal cancer" OR "Esophagea"OR "Esophageal tumor" OR "Stomach Neoplasms" OR "Stomach canceiNeoplasms" OR "Esophageal cancer"OR "stomach tumor" OR "gastric neoplasms" OR "gastic cancer" OR "gastic tumor" OR "Duodenal Neoplasms" oR "Duodenal cancer oR "Duodenal tumorOR "small bowel cancer""small intestine cancer " OR "small intestine tumor " OR "small intestineneoplasms " OR "Appendiceal"Appendiceal cancer" OR "Colonic Neoplasms" OR "Colonic cancer" OR "ColonicNeonlasnnsr" OR "Liver Neoplasms" OR "Liver cancer" OR "Liver tumor" OR "Gallbladdertumor" OR "Rectal Neoplasns" OR"RecNeoplasms" OR "Gallbladder cancer" OR "Gallbladder tumor" OR "Pancreatic Neoplasms" OR "Pancreatic cancer OR "Pancreatic tumor'".t. ab.kw |
| #4 | #1 AND #2 AND #3 |

**China National Knowledge Infrastructure: January 9, 2025**

**Search Strategy:**

| # | 检索策略 |
| --- | --- |
| 1 | (主题=消化系统 + 冑肠道 + 食管 + 冑 + 十ニ指肠 + 小肠 + 結肠 + 直肠 + 肝 + 胆嚢 + 阑尾) AND (主题=肿瘤 + 癌) AND (主题=胃肠道微牛物组 +肠道菌群 +菌群) AND (主题=系统评价 + meta + 荟萃) |

**China Biology Medicine disc: January 9, 2025**

**Search Strategy:**

| 序号 | 检索表达式 |
| --- | --- |
| 1) | ( "消化系统"[常用字段:智能] OR "胃肠道"[常用字段:智能] OR "食管"[常用字段:智能] OR "胃"[常用字段:智能] OR "十二指肠"[常用字段:智能] OR "小肠"[常用字段:智能] OR "结肠"[常用字段:智能] OR "直肠"[常用字段:智能] OR "肝"[常用字段:智能] OR "胆囊"[常用字段:智能] OR "阑尾"[常用字段:智能] OR "胰"[常用字段:智能]) AND( "肿瘤"[常用字段:智能] OR "癌"[常用字段:智能]) AND( "胃肠道微生物组"[常用字段:智能] OR "肠道菌群"[常用字段:智能] OR "菌群"[常用字段:智能]) AND( "系统评价"[常用字段:智能] OR "meta"[常用字段:智能] OR "荟萃分析"[常用字段:智能]) |

**Wanfang Data Knowledge Service Platform: January 9, 2025**

**Search Strategy:**

| # | 检索策略 |
| --- | --- |
| 1 | 主题:(消化系统OR胃肠道OR食管OR胃OR十ニ指肠OR小肠OR结肠) AND 主题:(肿瘤OR癌) AND 主题:(胃肠道微生物组OR肠道菌群OR菌群) AND 题名:( 系统评价 OR meta OR 荟萃) |

**Table S2 Included Articles**

| No. | Article Reference Information |
| --- | --- |
| #1 | Mohammad Aidid E, Shalihin MSE, Md Nor A, et al. Risk of colorectal cancer due to Streptococcus gallolyticus: a systematic review. Med J Malaysia. 2023;78(3):404-410. |
| #2 | Alhhazmi AA, Alhamawi RM, Almisned RM, et al. Gut Microbial and Associated Metabolite Markers for Colorectal Cancer Diagnosis. *Microorganisms*. 2023;11(8):2037. Published 2023 Aug 8. doi:10.3390/microorganisms11082037 |
| #3 | Amitay EL, Krilaviciute A, Brenner H. Systematic review: Gut microbiota in fecal samples and detection of colorectal neoplasms. *Gut Microbes*. 2018;9(4):293-307. doi:10.1080/19490976.2018.1445957 |
| #4 | Aprile F, Bruno G, Palma R, et al. Microbiota Alterations in Precancerous Colon Lesions: A Systematic Review. *Cancers (Basel)*. 2021;13(12):3061. Published 2021 Jun 19. doi:10.3390/cancers13123061 |
| #5 | Avuthu N, Guda C. Meta-Analysis of Altered Gut Microbiota Reveals Microbial and Metabolic Biomarkers for Colorectal Cancer. *Microbiol Spectr*. 2022;10(4):e0001322. doi:10.1128/spectrum.00013-22 |
| #6 | Borges-Canha M, Portela-Cidade JP, Dinis-Ribeiro M, Leite-Moreira AF, Pimentel-Nunes P. Role of colonic microbiota in colorectal carcinogenesis: a systematic review. *Rev Esp Enferm Dig*. 2015;107(11):659-671. doi:10.17235/reed.2015.3830/2015 |
| #7 | Casimiro-Soriguer CS, Loucera C, Peña-Chilet M, Dopazo J. Towards a metagenomics machine learning interpretable model for understanding the transition from adenoma to colorectal cancer. *Sci Rep*. 2022;12(1):450. Published 2022 Jan 10. doi:10.1038/s41598-021-04182-y |
| #8 | Costa CPD, Vieira P, Mendes-Rocha M, Pereira-Marques J, Ferreira RM, Figueiredo C. The Tissue-Associated Microbiota in Colorectal Cancer: A Systematic Review. *Cancers (Basel)*. 2022;14(14):3385. Published 2022 Jul 12. doi:10.3390/cancers14143385 |
| #9 | Dai Z, Coker OO, Nakatsu G, et al. Multi-cohort analysis of colorectal cancer metagenome identified altered bacteria across populations and universal bacterial markers. *Microbiome*. 2018;6(1):70. Published 2018 Apr 11. doi:10.1186/s40168-018-0451-2 |
| #10 | Drewes JL, White JR, Dejea CM, et al. High-resolution bacterial 16S rRNA gene profile meta-analysis and biofilm status reveal common colorectal cancer consortia. NPJ Biofilms Microbiomes. 2017;3:34. Published 2017 Nov 29. doi:10.1038/s41522-017-0040-3 |
| #11 | Eastmond AK, Shetty C, Rizvi SMHA, et al. A Systematic Review of the Gastrointestinal Microbiome: A Game Changer in Colorectal Cancer. Cureus. 2022;14(8): e28545. Published 2022 Aug 29. doi:10.7759/cureus.28545 |
| #12 | Gaab ME, Lozano PO, Ibañez D, et al. A Meta-Analysis on the Association of Colibactin-Producing pks+ Escherichia coli with the Development of Colorectal Cancer. *Lab Med*. 2023;54(1):75-82. doi:10.1093/labmed/lmac072 |
| #13 | Gao W, Gao X, Zhu L, et al. Multimodal metagenomic analysis reveals microbial single nucleotide variants as superior biomarkers for early detection of colorectal cancer. *Gut Microbes*. 2023;15(2):2245562. doi:10.1080/19490976.2023.2245562 |
| #14 | Gethings-Behncke C, Coleman HG, Jordao HWT, et al. *Fusobacterium nucleatum* in the Colorectum and Its Association with Cancer Risk and Survival: A Systematic Review and Meta-analysis. *Cancer Epidemiol Biomarkers Prev*. 2020;29(3):539-548. doi:10.1158/1055-9965.EPI-18-1295 |
| #15 | Güven Gülhan Ü, Nikerel E, Çakır T, Erdoğan Sevilgen F, Durmuş S. Species-level identification of enterotype-specific microbial markers for colorectal cancer and adenoma. *Mol Omics*. 2024;20(6):397-416. Published 2024 Jul 8. doi:10.1039/d4mo00016a |
| #16 | Herlo LF, Salcudean A, Sirli R, et al. Gut Microbiota Signatures in Colorectal Cancer as a Potential Diagnostic Biomarker in the Future: A Systematic Review. *Int J Mol Sci*. 2024;25(14):7937. Published 2024 Jul 20. doi:10.3390/ijms25147937 |
| #17 | Hussan H, Clinton SK, Roberts K, Bailey MT. *Fusobacterium*'s link to colorectal neoplasia sequenced: A systematic review and future insights. *World J Gastroenterol*. 2017;23(48):8626-8650. doi:10.3748/wjg.v23.i48.8626 |
| #18 | Kharofa J, Apewokin S, Alenghat T, Ollberding NJ. Metagenomic analysis of the fecal microbiome in colorectal cancer patients compared to healthy controls as a function of age. *Cancer Med*. 2023;12(3):2945-2957. |
| #19 | Liu H., Wu H., Bilegsaikhan E., et al. Differential expression of intestinal microbiota in colorectal cancer compared with healthy controls: A systematic review and meta-analysis. International Journal of Clinical and Experimental Medicine, 9(6), 10923-10930 |
| #20 | Mo Z, Huang P, Yang C, et al. Meta-analysis of 16S rRNA Microbial Data Identified Distinctive and Predictive Microbiota Dysbiosis in Colorectal Carcinoma Adjacent Tissue. *mSystems*. 2020;5(2):e00138-20. Published 2020 Apr 14. doi:10.1128/mSystems.00138-20 |
| #21 | Obón-Santacana M, Mas-Lloret J, Bars-Cortina D, et al. Meta-Analysis and Validation of a Colorectal Cancer Risk Prediction Model Using Deep Sequenced Fecal Metagenomes. *Cancers (Basel)*. 2022;14(17):4214. Published 2022 Aug 30. doi:10.3390/cancers14174214 |
| #22 | Ranjbar M, Salehi R, Haghjooy Javanmard S, et al. The dysbiosis signature of Fusobacterium nucleatum in colorectal cancer-cause or consequences? A systematic review. *Cancer Cell Int*. 2021;21(1):194. Published 2021 Apr 6. doi:10.1186/s12935-021-01886-z |
| #23 | Riveros Escalona MA, Poloni JF, Krause MJ, Dorn M. Meta-analyses of host metagenomes from colorectal cancer patients reveal strong relationship between colorectal cancer-associated species. *Mol Omics*. 2023;19(5):429-444. Published 2023 Jun 12. doi:10.1039/d3mo00021d |
| #24 | Shah MS, DeSantis TZ, Weinmaier T, et al. Leveraging sequence-based faecal microbial community survey data to identify a composite biomarker for colorectal cancer. *Gut*. 2018;67(5):882-891. |
| #25 | Sze MA, Schloss PD. Leveraging Existing 16S rRNA Gene Surveys To Identify Reproducible Biomarkers in Individuals with Colorectal Tumors. *mBio*. 2018;9(3):e00630-18. Published 2018 Jun 5. doi:10.1128/mBio.00630-18 |
| #26 | Tabowei G, Gaddipati GN, Mukhtar M, et al. Microbiota Dysbiosis a Cause of Colorectal Cancer or Not? A Systematic Review. *Cureus*. 2022;14(10):e30893. Published 2022 Oct 31. doi:10.7759/cureus.30893 |
| #27 | Thomas AM, Manghi P, Asnicar F, et al. Metagenomic analysis of colorectal cancer datasets identifies cross-cohort microbial diagnostic signatures and a link with choline degradation. *Nat Med*. 2019;25(4):667-678. doi:10.1038/s41591-019-0405-7 |
| #28 | Villar-Ortega P, Expósito-Ruiz M, Gutiérrez-Soto M, Ruiz-Cabello Jiménez M, Navarro-Marí JM, Gutiérrez-Fernández J. The association between Fusobacterium nucleatum and cancer colorectal: A systematic review and meta-analysis. *Enferm Infecc Microbiol Clin (Engl Ed)*. 2022;40(5):224-234. doi:10.1016/j.eimce.2022.02.007 |
| #29 | Wirbel J, Pyl PT, Kartal E, et al. Meta-analysis of fecal metagenomes reveals global microbial signatures that are specific for colorectal cancer. *Nat Med*. 2019;25(4):679-689. doi:10.1038/s41591-019-0406-6 |
| #30 | Yu L, Zhao G, Wang L, et al. A systematic review of microbial markers for risk prediction of colorectal neoplasia. *Br J Cancer*. 2022;126(9):1318-1328. doi:10.1038/s41416-022-01740-7 |
| #31 | Zwezerijnen-Jiwa FH, Sivov H, Paizs P, Zafeiropoulou K, Kinross J. A systematic review of microbiome-derived biomarkers for early colorectal cancer detection. *Neoplasia*. 2023;36:100868. doi:10.1016/j.neo.2022.100868 |
| #32 | Jiang P, Wu S, Luo Q, Zhao XM, Chen WH. Metagenomic Analysis of Common Intestinal Diseases Reveals Relationships among Microbial Signatures and Powers Multidisease Diagnostic Models. mSystems. 2021;6(3):e00112-21. Published 2021 May 4. doi:10.1128/mSystems.00112-21 |
| #33 | Wu YJ, Xiong JF, Zhan CN, Xu H. Gut microbiota alterations in colorectal adenoma-carcinoma sequence based on 16S rRNA gene sequencing: A systematic review and meta-analysis. *Microb Pathog*. 2024;195:106889. doi:10.1016/j.micpath.2024.106889 |
| #34 | Islam MZ, Tran M, Xu T, Tierney BT, Patel C, Kostic AD. Reproducible and opposing gut microbiome signatures distinguish autoimmune diseases and cancers: a systematic review and meta-analysis. *Microbiome*. 2022;10(1):218. Published 2022 Dec 9. doi:10.1186/s40168-022-01373-1 |
| #35 | van Vorstenbosch R, Cheng HR, Jonkers D, et al. Systematic Review: Contribution of the Gut Microbiome to the Volatile Metabolic Fingerprint of Colorectal Neoplasia. *Metabolites*. 2022;13(1):55. Published 2022 Dec 30. doi:10.3390/metabo13010055 |
| #36 | Trivedi Y, Bolgarina Z, Desai HN, et al. The Role of Gut Microbiome in Hepatocellular Carcinoma: A Systematic Review. *Cureus*. 2023;15(8):e43862. Published 2023 Aug 21. doi:10.7759/cureus.43862 |
| #37 | Lederer AK, Rasel H, Kohnert E, et al. Gut Microbiota in Diagnosis, Therapy and Prognosis of Cholangiocarcinoma and Gallbladder Carcinoma-A Scoping Review. *Microorganisms*. 2023;11(9):2363. Published 2023 Sep 21. doi:10.3390/microorganisms11092363 |
| #38 | Mattos VC, Nascimento FSD, Suzuki MO, et al. MICRObiota on BILIOpancreatic malignant diseases [MICROBILIO]: A systematic review. *Clinics (Sao Paulo)*. 2022;77:100101. Published 2022 Sep 16. doi:10.1016/j.clinsp.2022.100101 |
| #39 | Merali N, Chouari T, Sweeney C, et al. The microbial composition of pancreatic ductal adenocarcinoma: a systematic review of 16S rRNA gene sequencing. *Int J Surg*. 2024;110(10):6771-6799. Published 2024 Oct 1. doi:10.1097/JS9.0000000000001762 |
| #40 | Hong J, Fu Y, Chen X, et al. Gut microbiome changes associated with chronic pancreatitis and pancreatic cancer: a systematic review and meta-analysis. *Int J Surg*. 2024;110(9):5781-5794. Published 2024 Sep 1. doi:10.1097/JS9.0000000000001724 |
| #41 | Jankowski WM, Fichna J, Tarasiuk-Zawadzka A. A systematic review of the relationship between gut microbiota and prevalence of pancreatic diseases. *Microb Pathog*. 2025;199:107214. doi:10.1016/j.micpath.2024.107214 |
| #42 | Memba R, Duggan SN, Ni Chonchubhair HM, et al. The potential role of gut microbiota in pancreatic disease: A systematic review. *Pancreatology*. 2017;17(6):867-874. doi:10.1016/j.pan.2017.09.002 |
| #43 | Deng J, Huang Y, Yu K, Luo H, Zhou D, Li D. Changes in the gut microbiome of patients with esophageal cancer: A systematic review and meta-analysis based on 16S gene sequencing technology. *Microb Pathog*. 2024;193:106784. doi:10.1016/j.micpath.2024.106784 |
| #44 | Zhang L, Li D, Zhang Y, et al. Changes in the gut microbiota of esophageal carcinoma patients based on 16S rRNA gene sequencing: a systematic review and meta-analysis. *Front Oncol*. 2024;14:1366975. Published 2024 Aug 29. doi:10.3389/fonc.2024.1366975 |
| #45 | Vadhwana B, Tarazi M, Boshier PR, Hanna GB. Evaluation of the Oesophagogastric Cancer-Associated Microbiome: A Systematic Review and Quality Assessment. *Cancers (Basel)*. 2023;15(10):2668. Published 2023 May 9. doi:10.3390/cancers15102668 |
| #46 | Chen J, Nie S, Qiu X, et al. Leveraging existing 16S rRNA microbial data to identify diagnostic biomarker in Chinese patients with gastric cancer: a systematic meta-analysis. *mSystems*. 2023;8(5):e0074723. doi:10.1128/msystems.00747-23 |
| #47 | Li Y, Hu Y, Zhan X, et al. Meta-analysis reveals *Helicobacter pylori* mutual exclusivity and reproducible gastric microbiome alterations during gastric carcinoma progression. *Gut Microbes*. 2023;15(1):2197835. doi:10.1080/19490976.2023.2197835 |
| #48 | Liu C, Ng SK, Ding Y, et al. Meta-analysis of mucosal microbiota reveals universal microbial signatures and dysbiosis in gastric carcinogenesis. *Oncogene*. 2022;41(28):3599-3610. doi:10.1038/s41388-022-02377-9 |
| #49 | Wang Y, Wang Y, Han W, et al. Intratumoral and fecal microbiota reveals microbial markers associated with gastric carcinogenesis. *Front Cell Infect Microbiol*. 2024;14:1397466. Published 2024 Sep 17. doi:10.3389/fcimb.2024.1397466 |
| #50 | Xue FB, Xu YY, Wan Y, Pan BR, Ren J, Fan DM. Association of H. pylori infection with gastric carcinoma: a Meta analysis. *World J Gastroenterol*. 2001;7(6):801-804. doi:10.3748/wjg.v7.i6.801 |
| #51 | Yang J., Si Y., Zhang J., Liu X., et al. Meta-analysis of the gut microbiota alterations in patients with gastric cancer in China. Medicine in Microecology, 15. http://dx.doi.org/10.1016/j.medmic.2022.100069 |
| #52 | Yang Y, Ji R, Zhao X, et al. Alterations in Gastric Mucosal Microbiota in Gastric Carcinogenesis: A Systematic Review and Meta-Analysis. *Front Med (Lausanne)*. 2021;8:754959. Published 2021 Dec 3. doi:10.3389/fmed.2021.754959 |
| #53 | Xie YF, Chao LM, Wang XY, et al. Systematic review of gut microbiota diversity and composition in Chinese pancreatic cancer patients. Chin J Evid-based Med. 2023;23(2):186-190. doi:10.7507/1672-2531.202210030 |
| #54 | Zong LB, Wang JF, Sun JH. Meta analysis of intestinal flora and risk of colorectal cancer. Nat Sci J Harbin Norm Univ. 2018;(10):22-26. doi:10.3969/j.issn.1007-9831.2018.10.007 |
| #55 | Dong HX, Liang H. Meta-analysis of the association between Helicobacter pylori infection and colorectal cancer in populations from different countries. Med J Chin PLA. 2015;40(3):236-241. http://www--sinomed--ac--cn--https.sinomed.gzzyy.qfclo.com:2222/zh/detail.do?ui=2015556016 |
| #56 | Xu XF, Bi JP. Meta-analysis of the relationship between CagA^+ Hp infection and gastric cancer in Chinese population. Chin Trop Med,2006,(12):2122-2123. doi: 10.3969/j.issn.1009-9727.2006.12.008 |
| #57 | Tian WJ, Wang BY, Zhang JZ, et al. Meta-analysis of the relationship between Helicobacter pylori infection and gastric cancer in China. Chin J Evid-based Med. 2006;6(11):833-838,2006,(11):833-838. doi: 10.3969/j.issn.1672-2531.2006.11.011 |
| #58 | Hu HL, Dong SF. Meta-analysis of the relationship between Helicobacter pylori infection and gastric cancer. Chin Health Stat. 2006;6(1):65-67. doi:10.3969/j.issn.1002-3674.2006.01.021. |
| #59 | Liu AM, Zhao JK. Meta-analysis of the relationship between Helicobacter pylori infection and gastric cancer. Chin Oncol. 2006;6(9):583-586. doi:10.3969/j.issn.1004-0242.2006.09.004. |

**Table S3 Results of methodological quality evaluation**

| Author (Year) | Study eligibility criteria | | | | | | Identification and selection of studies | | | | | | Data collection and study appraisal | | | | | | Synthesis and findings | | | | | | | Risk Of Bias in the Review | | | |
| --- | --- | --- | --- | --- | --- | --- | --- | --- | --- | --- | --- | --- | --- | --- | --- | --- | --- | --- | --- | --- | --- | --- | --- | --- | --- | --- | --- | --- | --- |
|  | 1.1 | 1.2 | 1.3 | 1.4 | 1.5 | Concern | 2.1 | 2.2 | 2.3 | 2.4 | 2.5 | Concern | 3.1 | 3.2 | 3.3 | 3.4 | 3.5 | Concern | 4.1 | 4.2 | 4.3 | 4.4 | 4.5 | 4.6 | Concern | A | B | C | Concern |
| Deng et al. (2024) | PN | Y | PY | PY | PY | Low | PY | N | PY | Y | Y | Low | Y | Y | PY | Y | PY | Low | PY | PY | PY | Y | PY | PY | Low | Y | PY | Y | Low |
| Zhang et al. (2024) | Y | Y | Y | PY | PN | Low | PY | N | Y | Y | PY | Low | Y | Y | Y | Y | PY | Low | PY | Y | PY | PY | PY | Y | Low | Y | PY | Y | Low |
| Vadhwana et al. (2023) | PN | PY | PY | PY | PN | Low | Y | PY | PY | Y | PY | Low | NI | Y | NI | Y | Y | Low | PY | NI | Y | Y | PY | PY | Low | Y | PY | Y | Low |
| Chen et al. (2023) | PN | PN | PN | NI | NI | High | PN | N | PY | Y | NI | High | NI | PN | PY | N | NI | High | Y | NI | PY | PY | PY | N | Low | PN | PY | PY | Low |
| Li et al. (2023) | PN | PN | PN | NI | NI | High | PN | N | PY | Y | NI | High | NI | PN | PY | N | NI | High | Y | NI | PY | PY | PY | N | Low | PN | PY | Y | Low |
| Liu et al. (2022) | PN | PN | PN | NI | NI | High | NI | NI | NI | NI | NI | Unclear | NI | PN | PY | N | NI | High | Y | PY | PY | PY | PY | NI | Low | PN | PY | Y | Low |
| Wang et al. (2024) | PN | PY | PY | PY | PN | Low | PY | N | Y | N | Y | High | NI | NI | PY | NI | NI | Unclear | Y | PY | PY | PY | PY | NI | Low | PN | PY | Y | Low |
| Xue et al. (2001) | PN | NI | NI | NI | NI | Unclear | PN | N | PN | Y | NI | High | NI | NI | PY | NI | NI | Unclear | Y | NI | PY | PY | PY | N | Low | PN | PY | Y | Low |
| Yang et al. (2023) | PN | PY | PY | PY | PY | Low | Y | Y | PY | PY | Y | Low | Y | PY | PY | Y | NI | Low | Y | PN | PY | PY | PY | PY | Low | Y | PY | Y | Low |
| Yang et al. (2021) | Y | Y | PY | PY | PY | Low | Y | Y | PY | Y | Y | Low | Y | Y | PY | Y | PY | Low | Y | Y | Y | Y | PY | PY | Low | Y | PY | Y | Low |
| Hu et al. (2006) | PN | PY | PY | PY | PY | Low | PN | Y | NI | PY | NI | Low | NI | PN | PY | NI | NI | Unclear | Y | PY | Y | Y | Y | N | Low | PN | PY | PY | Low |
| Liu et al. (2006) | PN | PY | PY | PY | PN | Low | PY | N | NI | PY | NI | High | NI | NI | NI | NI | NI | Unclear | Y | PY | PY | PY | PN | N | Low | PN | PY | PY | Low |
| Tian et al. (2006) | PN | PY | PN | PY | PY | Low | PN | N | NI | PY | NI | High | NI | PY | PY | NI | NI | Unclear | Y | PN | Y | Y | PY | N | Low | PY | PY | Y | Low |
| Xu et al. (2006) | PN | PY | PY | PN | PY | High | PN | Y | NI | PY | NI | High | NI | NI | PY | NI | NI | Unclear | Y | PY | PY | PY | PY | N | Low | PN | PY | PY | Low |
| Aidid et al. (2023) | PN | PY | Y | PY | Y | Low | PY | N | PY | PN | PN | Low | PY | PY | PY | PN | PN | Low | Y | PY | Y | Y | PY | PY | Low | Y | PY | Y | Low |
| Alhhazmi et al. (2023) | PN | PY | PY | PY | PY | Low | PY | Y | PY | Y | Y | Low | PY | Y | PY | Y | Y | Low | Y | N | PY | Y | PY | PY | High | PY | PY | Y | Low |
| Amitay et al. (2018) | PN | PY | PY | PN | PY | Low | PY | Y | PN | PN | Y | Low | NI | PY | NI | Y | Y | Low | PY | N | PY | Y | PY | PY | High | PY | PY | PY | Low |
| Aprile et al. (2021) | PN | PN | PN | PY | PN | Low | PN | Y | PN | PY | Y | Low | NI | PN | PY | N | NI | High | PY | N | PY | Y | PY | N | High | PN | PY | PY | Low |
| Avuthu et al. (2022) | NI | NI | NI | NI | NI | Unclear | NI | NI | NI | NI | NI | Unclear | NI | NI | PY | NI | NI | Unclear | NI | PY | PY | PY | PY | NI | Low | N | PY | PY | High |
| Borges et al. (2015) | PN | PY | PN | PY | PN | Low | PN | N | PY | Y | NI | Low | NI | PN | PY | NI | NI | Unclear | PY | N | PY | Y | PY | N | Low | PN | PY | PY | Low |
| Casimiro et al. (2022) | NI | NI | NI | NI | NI | Unclear | NI | NI | NI | NI | NI | Unclear | NI | NI | PY | NI | NI | Unclear | NI | PY | PY | PY | PY | NI | Low | N | PY | PY | High |
| Costa et al. (2022) | PN | PY | PY | PY | PN | Low | PY | N | PN | Y | Y | Low | Y | Y | PY | Y | Y | Low | PY | PN | PY | PY | PY | PY | Low | PY | PY | PY | Low |
| Dai et al. (2018) | NI | NI | NI | NI | NI | Unclear | NI | NI | NI | NI | NI | Unclear | NI | NI | PY | NI | NI | Unclear | NI | PY | PY | PY | PY | NI | Low | N | PY | PY | High |
| Drewes et al. (2017) | NI | NI | NI | NI | NI | Unclear | NI | NI | NI | NI | NI | Unclear | NI | PY | PY | NI | NI | Unclear | NI | PY | PY | PY | PY | NI | Low | N | PY | PY | High |
| Eastmond et al. (2022) | PN | PN | PN | PY | PN | High | PY | Y | PY | PN | NI | Low | NI | PY | PY | Y | NI | Low | PY | N | PY | Y | PY | Y | Low | PY | PY | PY | Low |
| Gaab et al. (2023) | PN | PY | Y | PY | PN | Low | Y | Y | PY | Y | NI | Low | Y | PY | PY | Y | NI | Low | PY | PN | PY | Y | Y | PY | Low | Y | PY | PY | Low |
| Gao et al. (2023) | NI | NI | NI | NI | NI | Low | NI | NI | NI | NI | NI | Unclear | NI | NI | PY | NI | NI | Unclear | NI | PY | PY | PY | PY | N | Low | N | PY | PY | High |
| Gethings et al. (2020) | PN | PY | PY | Y | Y | Low | Y | N | PY | Y | Y | Low | Y | Y | PY | Y | NI | Low | PY | PN | PY | PY | PY | PY | Low | PY | Y | PT | Low |
| Gülhan et al. (2024) | NI | NI | NI | NI | NI | Unclear | NI | NI | NI | NI | NI | Unclear | NI | NI | PY | NI | NI | Unclear | NI | PY | PY | PY | PY | N | Low | PN | PY | PY | Low |
| Herlo et al. (2024) | PN | PY | PY | PY | PN | Low | PN | Y | NI | PN | NI | High | NI | PY | PY | N | NI | High | PY | PN | PY | PY | PY | N | Low | PY | PY | PY | Low |
| Hussan et al. (2017) | PN | PY | PY | PY | PN | Low | Y | N | PY | PY | Y | Low | NI | PY | PY | N | NI | High | PY | N | PY | Y | PY | N | Low | PN | PY | PY | Low |
| Kharofa et al. (2022) | NI | NI | NI | NI | NI | Unclear | NI | NI | NI | NI | NI | Unclear | NI | NI | PY | NI | NI | Unclear | NI | PY | PY | PY | PY | N | Low | PY | PY | PY | Low |
| Liu et al. (2016) | PN | PY | PY | PY | PY | Low | PN | Y | PY | Y | NI | Low | Y | Y | PY | PY | NI | Low | PY | PN | PY | PY | PY | PY | Low | Y | PY | PY | Low |
| Mo et al. (2020) | NI | NI | NI | NI | NI | Unclear | NI | NI | NI | NI | NI | Unclear | NI | NI | PY | NI | NI | Unclear | NI | PY | PY | PY | PY | N | Low | PY | PY | PY | Low |
| Obón et al. (2022) | PN | PN | PN | PN | NI | Low | NI | NI | PN | PY | Y | Low | NI | PY | PY | NI | NI | Unclear | PY | PY | PY | PY | Y | N | Low | PY | PY | PY | Low |
| Ranjbar et al. (2021) | PN | PY | PY | PY | PN | Low | Y | Y | PY | Y | NI | Low | NI | PY | PY | NI | NI | Unclear | PY | N | PY | Y | PY | N | Low | PY | PY | PY | Low |
| Riveros et al. (2023) | NI | NI | NI | NI | NI | Unclear | NI | NI | NI | NI | NI | Unclear | NI | NI | PY | NI | NI | Unclear | NI | PY | PY | PY | PY | N | Low | PY | PY | PY | Low |
| Shah et al. (2018) | PN | PY | PY | Y | PY | Low | PN | PY | PN | Y | NI | Low | NI | Y | PY | NI | NI | Unclear | Y | PY | PY | PY | PY | N | Low | PY | PY | PY | Low |
| Sze et al. (2018) | NI | NI | NI | NI | NI | Unclear | NI | NI | NI | NI | NI | Unclear | NI | NI | PY | NI | NI | Unclear | NI | PY | PY | PY | PY | N | Low | PY | Y | PY | Low |
| Tabowei et al. (2022) | PN | PY | PY | PN | PN | High | Y | N | PN | PN | Y | High | NI | PN | PY | Y | NI | High | PY | N | PY | Y | PY | PY | Low | Y | PY | Y | Low |
| Thomas et al. (2019) | NI | NI | PN | NI | NI | Unclear | NI | NI | NI | NI | NI | Unclear | NI | NI | PY | NI | NI | Unclear | NI | PN | PN | PY | PY | N | High | N | PY | PY | High |
| Villar et al. (2022) | PN | PY | PN | PN | PN | High | PY | PN | PN | PN | NI | High | NI | PY | PY | NI | NI | Unclear | PY | PN | PY | PN | PY | N | High | PY | PY | PY | Low |
| Wirbel et al. (2019) | PN | PY | PN | PY | PY | Low | PN | N | PN | Y | NI | High | NI | NI | PY | NI | NI | Unclear | NI | PY | PY | PY | PY | N | Low | N | PY | PY | High |
| Yu et al. (2022) | Y | Y | Y | PY | Y | Low | Y | Y | PY | Y | Y | Low | NI | PY | PY | Y | NI | Low | PY | Y | PY | PY | PY | PY | Low | PY | PY | PY | Low |
| Zwezerijnen et al. (2023) | PN | PY | PY | PY | PY | Low | Y | N | PY | Y | Y | Low | Y | Y | PY | Y | Y | Low | PY | N | PY | Y | PY | PY | Low | PY | PY | PY | Low |
| Jiang et al. (2021) | PN | PN | PN | PN | PN | High | PY | N | PY | PY | NI | Low | NI | NI | PY | NI | NI | Unclear | NI | PY | PY | PY | PY | N | Low | PN | PY | PY | Low |
| Wu et al. (2024) | Y | Y | Y | PY | PN | Low | Y | N | PY | Y | NI | Low | NI | PY | PY | Y | PY | Low | PY | PY | PY | PY | PY | PY | Low | PY | PY | PY | Low |
| Dong et al. (2015) | PN | PY | PY | PY | PY | Low | Y | N | PY | Y | NI | Low | NI | PN | PY | NI | NI | Unclear | PY | PN | PY | PY | PY | N | Low | PN | PY | PY | Low |
| Zong et al. (2018) | PN | PY | PY | PY | PN | Low | PN | NI | NI | NI | NI | Unclear | NI | NI | PY | PN | NI | Unclear | PY | PY | PY | PN | PN | PN | High | N | PY | PY | High |
| Islam et al. (2022) | PN | PY | PY | PY | PN | Low | Y | N | PY | Y | Y | Low | Y | Y | PY | Y | NI | Low | PY | PY | PY | PY | PY | PY | Low | PY | PY | PY | Low |
| Huybrechtset al. (2023) | Y | PN | PY | PN | PY | High | Y | Y | PY | PY | Y | Low | Y | NI | PY | Y | Y | Low | PY | Y | PY | Y | PY | PY | Low | PN | PY | PY | Low |
| Trivedi et al. (2023) | PN | PN | PN | PY | PN | High | Y | N | PN | PN | NI | High | NI | PN | PY | Y | NI | Low | PN | N | PY | Y | PY | PY | Low | Y | Y | PY | Low |
| Lederer et al. (2023) | PN | PY | PN | Y | Y | Low | Y | Y | PY | PN | NI | Low | NI | PY | PY | NI | NI | Unclear | PY | N | PY | Y | PY | N | High | PN | PY | PY | Low |
| Mattos et al. (2022) | Y | PY | PY | Y | PN | Low | Y | Y | PY | Y | NI | Low | Y | Y | Y | Y | NI | Low | PY | Y | PY | Y | PY | PY | Low | Y | Y | Y | Low |
| Merali et al. (2024) | Y | PY | PY | Y | Y | Low | Y | Y | PY | Y | Y | Low | Y | PY | PY | Y | NI | Low | PY | Y | PY | Y | PY | PY | Low | PY | PY | PY | Low |
| Hong et al. (2024) | Y | PN | PY | PN | PN | High | Y | Y | PY | Y | Y | Low | PY | PY | PY | Y | NI | Low | Y | Y | Y | PY | Y | PY | Low | PN | PY | PY | Low |
| Jankowski et al. (2024) | PN | PY | PY | PY | PN | Low | Y | N | PY | N | Y | High | NI | PY | PY | NI | NI | Unclear | Y | N | PY | Y | PN | N | High | N | PY | PY | High |
| Memba et al. (2017) | PN | PN | PN | PN | PN | High | Y | Y | PY | Y | NI | Low | NI | PY | PY | Y | NI | Low | Y | N | PY | Y | PY | PY | Low | PN | PY | PY | Low |
| Xie et al. (2023) | PN | PY | PY | PY | PN | Low | Y | Y | PY | PY | Y | Low | Y | PY | PY | Y | Y | Low | Y | PN | PY | PY | PY | PY | Low | Y | Y | Y | Low |
