# Supplementary figures and images for: Characterization of dysbiosis patterns in gut microbiota of digestive system cancers: an umbrella review
Source: Front Microbiol. 2026 Apr 28;17:1782471. doi: 10.3389/fmicb.2026.1782471 (PMC13161176; doi:10.3389/fmicb.2026.1782471)

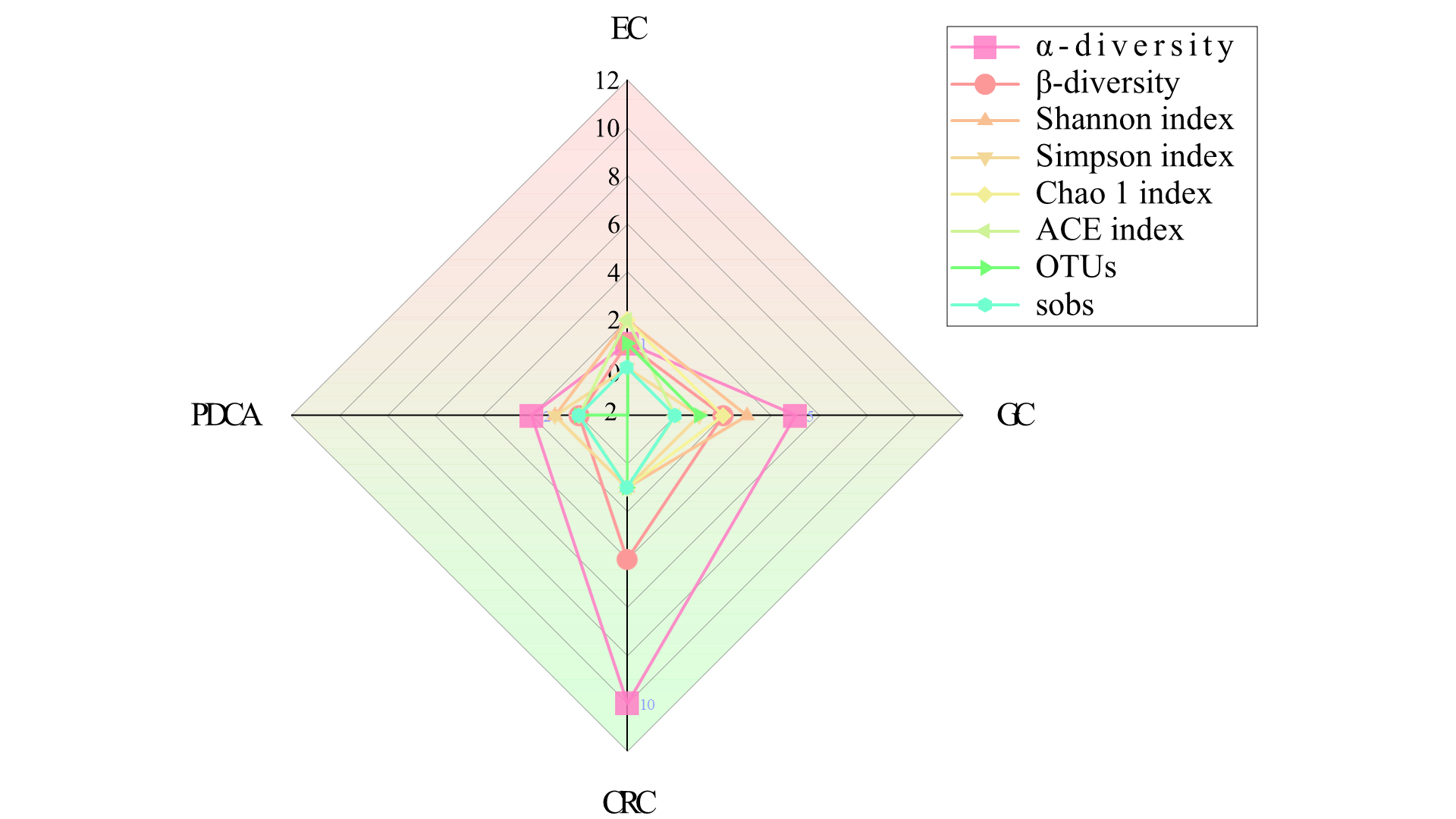

Supplement: Supplementary file 3 [file Image_1.tiff]

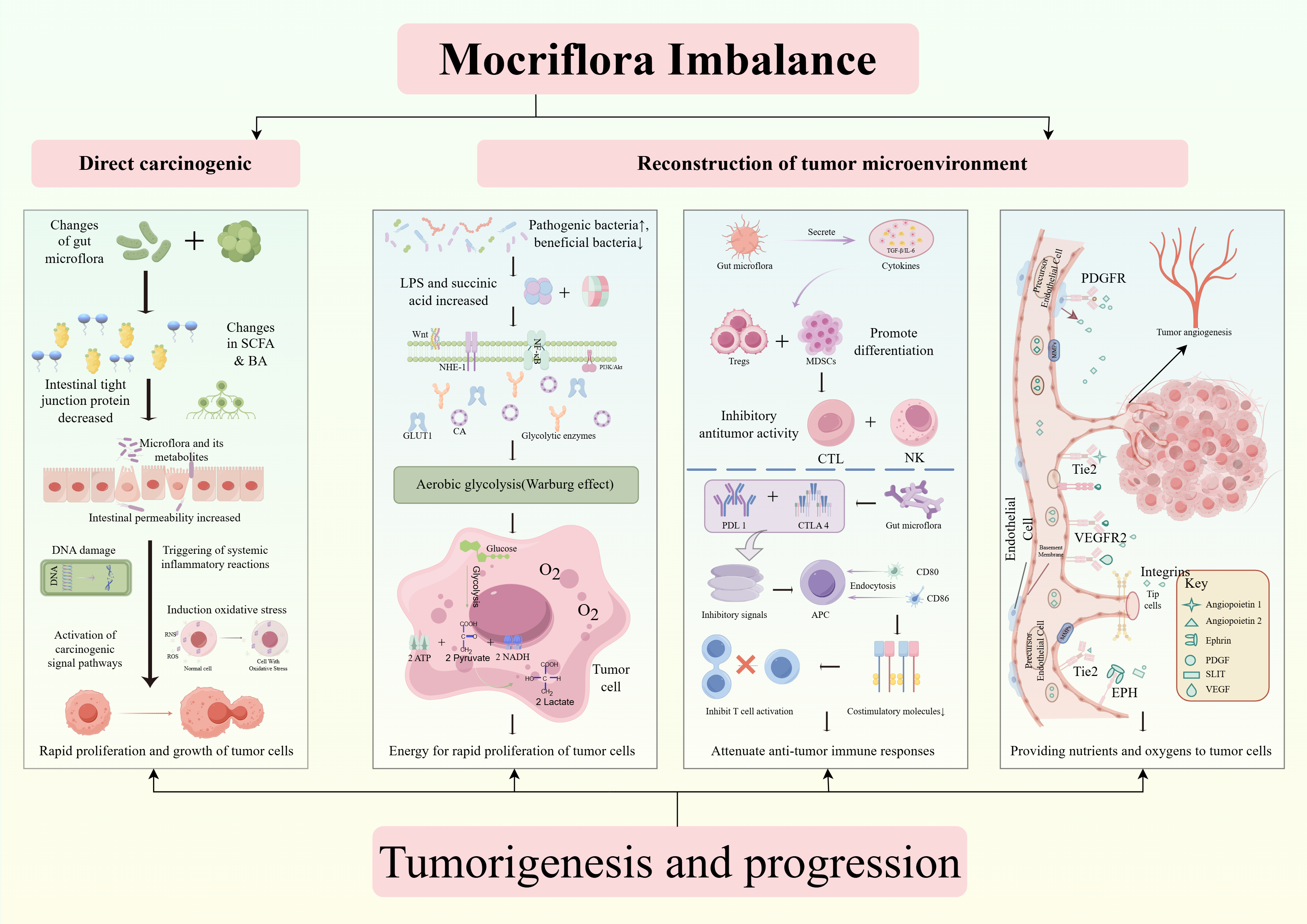

Supplement: Supplementary file 4 [file Image_2.tif]
